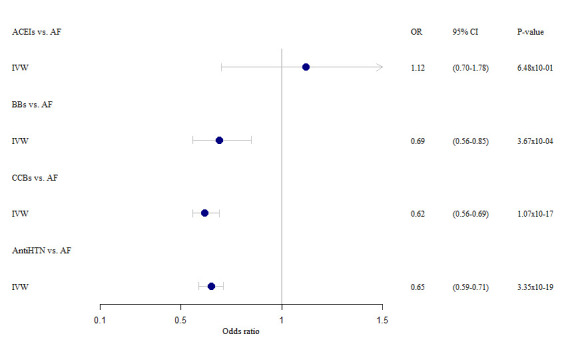# Antihypertensive Drugs for the Prevention of Atrial Fibrillation: A Drug Target Mendelian Randomization Study

**DOI:** 10.1161/HYPERTENSIONAHA.123.21858

**Published:** 2024-06-19

**Authors:** Sven Geurts, Martijn J. Tilly, Zuolin Lu, Bruno H.C. Stricker, Jaap W. Deckers, Natasja M.S. de Groot, Clint L. Miller, M. Arfan Ikram, Maryam Kavousi

**Affiliations:** 1 Department of Epidemiology (S.G., M.J.T., Z.L., B.H.C.S., J.W.D., M.A.I., M.K.), Erasmus MC, University Medical Center Rotterdam, The Netherlands.; 2 Department of Cardiology (N.M.S.G.), Erasmus MC, University Medical Center Rotterdam, The Netherlands.; 3 Department of Biochemistry and Molecular Genetics, University of Virginia, Charlottesville (C.L.M.).

**Keywords:** antihypertensive agents, atrial fibrillation, drug repositioning, epidemiology, genomics, Mendelian randomization analysis, polymorphism, single nucleotide

## Abstract

**BACKGROUND:**

We investigated the potential impact of antihypertensive drugs for atrial fibrillation (AF) prevention through a drug target Mendelian randomization study to avoid the potential limitations of clinical studies.

**METHODS:**

Validated published single-nucleotide polymorphisms (SNPs) that mimic the action of 12 antihypertensive drug classes, including alpha-adrenoceptor blockers, adrenergic neuron blockers, angiotensin-converting enzyme inhibitors, angiotensin-II receptor blockers, beta-adrenoceptor blockers, centrally acting antihypertensive drugs, calcium channel blockers, loop diuretics, potassium-sparing diuretics and mineralocorticoid receptor antagonists, renin inhibitors, thiazides and related diuretic agents, and vasodilators were used. We estimated, via their corresponding gene and protein targets, the downstream effect of these drug classes to prevent AF via systolic blood pressure using 2-sample Mendelian randomization analyses. The SNPs were extracted from 2 European genome-wide association studies for the drug classes (n=317 754; n=757 601) and 1 European genome-wide association study for AF (n=1 030 836).

**RESULTS:**

Drug target Mendelian randomization analyses supported the significant preventive causal effects of lowering systolic blood pressure per 10 mm Hg via alpha-adrenoceptor blockers (n=11 SNPs; odds ratio [OR], 0.34 [95% CI, 0.21–0.56]; *P*=2.74×10^−05^), beta-adrenoceptor blockers (n=17 SNPs; OR, 0.52 [95% CI, 0.35–0.78]; *P*=1.62×10^−03^), calcium channel blockers (n=49 SNPs; OR, 0.50 [95% CI, 0.36–0.70]; *P*=4.51×10^−05^), vasodilators (n=19 SNPs; OR, 0.53 [95% CI, 0.34–0.84]; *P*=7.03×10^−03^), and all 12 antihypertensive drug classes combined (n=158 SNPs; OR, 0.64 [95% CI, 0.54–0.77]; *P*=8.50×10^−07^) on AF risk.

**CONCLUSIONS:**

Our results indicated that lowering systolic blood pressure via protein targets of various antihypertensive drugs seems promising for AF prevention. Our findings inform future clinical trials and have implications for repurposing antihypertensive drugs for AF prevention.

**NOVELTY AND RELEVANCE**

**What Is New?**

This study provides insights into the causal effects of antihypertensive drugs on the risk of atrial fibrillation using a drug target Mendelian randomization approach by using validated genetic variants that mimic the effects of 12 antihypertensive drug classes.

**What Is Relevant?**

Atrial fibrillation is the most common arrhythmia worldwide. Hypertension is the main modifiable risk factor for atrial fibrillation prevention, but large-scale randomized clinical trials that investigate the primary prevention of atrial fibrillation using antihypertensive drugs are lacking. Mendelian randomization is a powerful statistical method to assess the causality of associations, which may be more difficult via conventional randomized clinical trials.

**Clinical/Pathophysiological Implications?**

The current study supports the causal effect of several antihypertensive drugs on atrial fibrillation. This study extends the knowledge regarding the preventive impact of antihypertensive drugs on atrial fibrillation risk. These findings inform future clinical trials and could have implications for repurposing antihypertensive drugs to prevent atrial fibrillation.

Atrial fibrillation (AF) is the most common cardiac arrhythmia globally and is markedly associated with high morbidity, mortality, and a significant burden on patients and health care.^1^ The mechanisms underlying AF are not yet entirely understood. However, previous studies have indicated that AF may in part be a manifestation of hypertensive heart disease or hypertensive target organ damage, which highlights the critical role of managing hypertension to prevent AF development.^1–3^

As a complex and polygenic disease, it has been challenging to develop effective preventive therapies for AF. However, given the role of hypertension as a risk factor for AF, the potential of antihypertensive drugs to prevent AF remains biologically plausible.^2–5^ Nonetheless, earlier studies investigating the additive value of antihypertensive drugs in AF prevention were mainly retrospective, observational in nature, post hoc analyses, conducted in selected AF patients with underlying heart disease, or only investigated a specific antihypertensive drug.^4,5^ Although the post hoc analyses from the SPRINT (Systolic Blood Pressure Intervention Trial) suggest intensive treatment to be promising in reducing the risk of AF in hypertensive patients,^6^ large randomized clinical trials (RCTs) and comprehensive meta-analyses investigating the role of blood pressure reduction through various antihypertensive drugs for AF prevention are so far lacking. This is not surprising, as RCTs that investigate (new) drugs are challenging, costly, often restricted to older or high-risk patients, and have a relatively short duration of follow-up.

Mendelian randomization (MR) has emerged as a powerful statistical method to assess the causality of associations, which may be more difficult via conventional RCTs.^7^ By exploiting drug target-related genetic variants that mimic the effects of drugs, MR studies prove a valid and reliable alternative to RCTs to assess the effects of existing and new drugs to prevent disease.^8,9^

We therefore aimed to investigate the effects of 12 separate antihypertensive drug classes: alpha-adrenoceptor blockers, adrenergic neuron blockers, angiotensin-converting enzyme inhibitors, angiotensin-II receptor blockers, beta-adrenoceptor blockers, centrally acting antihypertensive drugs, calcium channel blockers, loop diuretics, potassium-sparing diuretics and mineralocorticoid receptor antagonists, renin inhibitors, thiazides and related diuretic agents, vasodilators, and their combined effect on AF risk using a drug target MR approach.

## METHODS

Anonymized data and materials have been made publicly available online and can be accessed at https://academic.oup.com/ije/article/49/4/1132/5537361?login=true#supplementary-data and http://csg.sph.umich.edu/willer/public/afib2018/.

This study complies with the Declaration of Helsinki and has been conducted using publicly available summary statistics from multiple studies.^10,11^ The summary statistics from the study on antihypertensive drugs^11^ are available at https://academic.oup.com/ije/article/49/4/1132/5537361?login=true#supplementary-data. The summary statistics from the genome-wide association study (GWAS) meta-analysis on AF^10^ are available at http://csg.sph.umich.edu/willer/public/afib2018/. No original data were collected for this drug target MR study. Ethical approval and informed consent from each participant for each of the studies included in the current investigation can be found in the original publications.^10,11^ The analysis of anonymous, publicly available summary statistics did not require additional ethical approval; therefore, the requirement for informed consent was waived.

### Antihypertensive Drug Exposures

Recent evidence showed that genetic proxies for the effect of antihypertensive drug classes can be used to assess the effect of antihypertensive drugs on diseases using a drug target MR approach.^11,12^ We adopted the 2 methods used by 2 prior studies to evaluate the effect of antihypertensive drugs on AF risk.^11,12^

First, we extracted published genetic variants or single-nucleotide polymorphisms (SNPs) associated with the effect of 12 antihypertensive drug classes among users, including alpha-adrenoceptor blockers, adrenergic neuron blockers, angiotensin-converting enzyme inhibitors, angiotensin-II receptor blockers, beta-adrenoceptor blockers, centrally acting antihypertensive drugs, calcium channel blockers, loop diuretics, potassium-sparing diuretics and mineralocorticoid receptor antagonists, renin inhibitors, thiazides and related diuretic agents, and vasodilators found by Walker et al.^11^ In short, Walker et al^11^ used drug substance information from the aforesaid antihypertensive drug classes to identify their corresponding target genes and pharmacologically active protein targets in the DrugBank database (https://go.drugbank.com/). The Genotype-Tissue Expression project database,^13^ which contains expression quantitative trait loci analyses of 48 tissues from 714 donors, was then used to identify the best genetic variants as instruments for each corresponding protein target. Subsequently, the association between the antihypertensive drug genetic variants and systolic blood pressure (SBP) was validated by estimating their effect on SBP using a 2-sample MR analysis. Genetic variants that showed an effect on SBP were retained in their analyses. A total of 293 genetic variants were nominally significantly (*P*<0.05) associated with SBP. The published antihypertensive drug genetic variants associated with protein-coding target genes for these drugs were used as individual as well as combined exposures in our drug target MR study. The GWAS meta-analysis of SBP that Walker et al^11^ used to conduct their 2-sample MR to validate their genetic instruments was based on data from the UK Biobank cohort.^13^ This GWAS by Neale et al^14^ encompassed n=317 754 participants from European descent (n=317 754, 100%).

Next, as secondary or complementary analyses, we extracted published genetic variants associated with 3 antihypertensive drug classes (angiotensin-converting enzyme inhibitors, beta-adrenoceptor blockers, and calcium channel blockers identified by Gill et al).^12^ In essence, Gill et al^12^ also used the substance information of the aforementioned antihypertensive drugs to identify target genes and proteins using the DrugBank database (https://go.drugbank.com/). Then the GeneCards platform^15^ was used to identify genetic variants within genomic regions that correspond to the genes, promoters, and/or enhancers of interest. The GeneCards platform is a novel database of human enhancers and their target genes that mines data from 4 sources: Ensembl, FANTOM5, VISTA, and ENCODE.^15^ A total of 31 genetic variants were genome-wide significantly (*P*<5×10^−08^) associated with SBP. Again, we used these published antihypertensive drug genetic variants as individual and combined exposures in our study to complement our initial analyses. The GWAS meta-analysis of SBP that Gill et al^12^ used to conduct their 2-sample MR to validate their genetic instruments was from the UK Biobank and the International Consortium of Blood Pressure.^16^ This GWAS by Evangelou et al^14^ encompassed n=757 601 participants from European descent (n=757 601, 100%). The baseline characteristics of both studies are depicted in Supplemental Methods S1.^11–17^

### AF Outcome

The GWAS by Nielsen et al^10^ encompassed 1 030  836 participants (60 620 AF cases [5.8%] and 970 216 controls) from European descent. The median age was not provided, and 53% were women.^10^ A total of 111 genetic variants were genome-wide significantly associated with AF. The AF genetic variants implicated genes that are expressed within the heart and have been suggested to affect cardiac development, cardiac ion channels, cardiac calcium signaling, structural integrity of the heart, and skeletal muscles.^10^ The summary statistics of these AF genetic variants were used as an outcome in our MR analyses. The baseline characteristics are shown in Supplemental Methods S2.^10,18^

### MR Analyses

Two-sample MR analyses were conducted to evaluate the effect of 12 separate antihypertensive drug classes and their combined effect on AF risk. Three assumptions should be considered and fulfilled for MR to provide valid and reliable causal estimates. The first assumption is that the genetic variant is strongly associated with the exposure of interest. The second assumption is that the genetic variant must only affect the outcome through its effect on the exposure. Lastly, the third assumption is that the genetic variant is not associated with any confounders of the exposure-outcome association. We extracted and selected published genetic variants that were nominally significantly associated with the trait of interest in 1 study and genome-wide significantly associated with the trait of interest in the second study. Next, we clumped the genetic variants to ensure that the instrumental variables for the exposure were independent (*P*<5.0×10^−02^ and r^2^<0.1) to avoid the use of correlated genetic variants that are in linkage disequilibrium.^7,19^ In addition, palindromic genetic variants were removed during the harmonization of the genetic variants. Moreover, European-ancestry genetic variants and summary statistics were selected in the subsequent MR analyses to avoid possible bias due to population stratification.^7,19^

The F statistic of each genetic instrument, a measure of strength to reduce instrument bias, was extracted from the original publications. Genetic variants with sufficient strength (F statistic >10) were included.^20^ We used the “TwoSampleMR” package^20,21^ to combine the effects of the individual and combined genetic variants on the exposure and outcome using the inverse-variance weighted (IVW) method.^22^ The IVW method was our main MR method and is a meta-analysis of all the Wald ratios from the individual genetic variants on the exposure and outcome. In other words, the IVW method represents a weighted mean estimate of the effect of genetically determined antihypertensive drugs on AF risk. In addition, we used the random effect IVW method to account for possible heterogeneity between genetic variants.

MR estimates are presented as odds ratios (ORs) with corresponding 95% CIs. Statistical significance was considered at a 2-sided *P*<0.05. We additionally presented the Bonferroni-corrected statistical significance of *P*<0.05/13 (3.85×10^−03^) to account for multiple testing. Data management and all statistical analyses were done using R statistical software (R 4.0.2: R Foundation for Statistical Computing, Vienna, Austria).

### MR Sensitivity Analyses

The rationale, assumptions, and sensitivity analyses are depicted in detail in Supplemental Methods S3.^17,23–27^ We estimated and depicted the amount of sample overlap in Supplemental Methods S1 and S2. In addition, we also estimated the amount of bias and type 1 error rate inflation due to sample overlap using an online calculator available at https://sb452.shinyapps.io/overlap/.^17^ More details regarding the estimated potential overlap are provided in Supplemental Methods S1 through S4.^10–18,22–27^ Additionally, we reran our analyses using more conservative r^2^ thresholds (0.01 and 0.001 instead of 0.1) to evaluate if our results are robust for all antihypertensive drug classes combined analyses for the primary and secondary analyses. Furthermore, we conducted more sensitivity analyses by also using FinnGen^28^ as an AF outcome data set to evaluate if our primary and secondary results were robust by further limiting potential sample overlap.

## RESULTS

### MR Analyses

**Figure 1.**
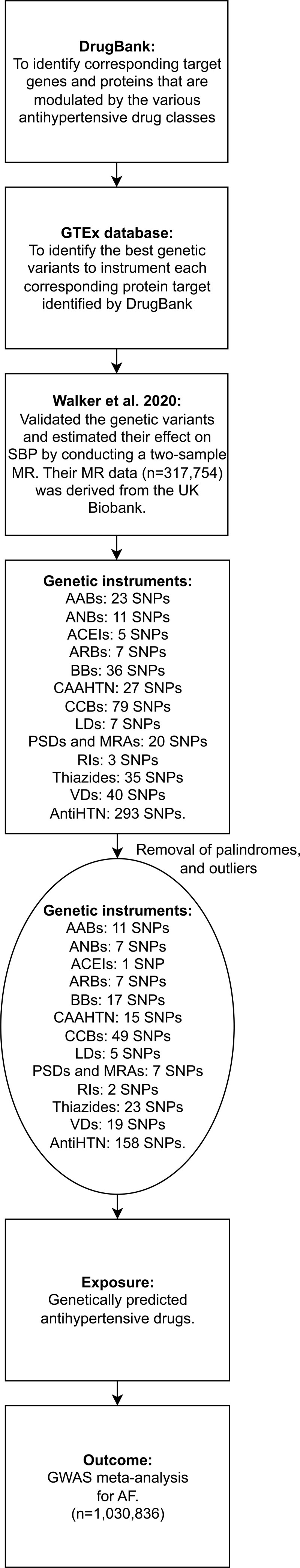
**Flow chat for selection of genetic variants for the primary analyses.** AABs indicates adrenergic neuron blockers; ACEIs, angiotensin-converting enzyme inhibitors; AF, atrial fibrillation; ANBs, alpha-adrenocepter blockers; AntiHTN; all 12 antihypertensive drug classes combined; ARBs, angiotensin-II receptor antagonists; BBs, beta-adrenoceptor blockers; CAAHTN, centrally acting antihypertensives; CCBs, calcium channel blockers; GTEx, Genotype-Tissue Expression; GWAS, genome-wide association study; LDs, loop diuretics; MR, Mendelian randomization; MRAs, mineralocorticoid receptor antagonists; n, number; PSDs, potassium-sparing diuretics and aldosterone antagonists; RIs, renin inhibitors; SBP, systolic blood pressure; SNP(s), single-nucleotide polymorphism(s); Thiazides, thiazides and related diuretics; and VDs, vasodilators.

In short, from our first data source, a total of 293 nominally significant genetic variants were associated with 12 classes of antihypertensive drugs for our primary analyses.^11^ For the secondary analyses, we included a total of 31 genome-wide significant genetic variants from the second data source.^12^ A total of 111 genome-wide significant genetic variants were associated with AF.^10^ The selection of genetic variants for the primary and secondary analyses is visually illustrated in detail in Figure 1 and Figure S1.

We clumped all genetic variants and excluded palindromes. Additionally, we excluded potential outliers by using the MR pleiotropy residual sum and outlier (MR-PRESSO) test and evaluated the sensitivity plots to select our genetic variants. This resulted in a total of 158 genetic variants available in the AF GWAS by Nielsen et al^10^ that were associated with AF and ≥1 classes of antihypertensive drugs in our primary analyses (Figure 1). These variants were subsequently used as instrumental variables or genetic instruments in our primary MR analyses. Similarly, we retained all 31 genetic variants from the Gill et al^12^ data set for our secondary analyses (Figure S1).

**Figure 2.**
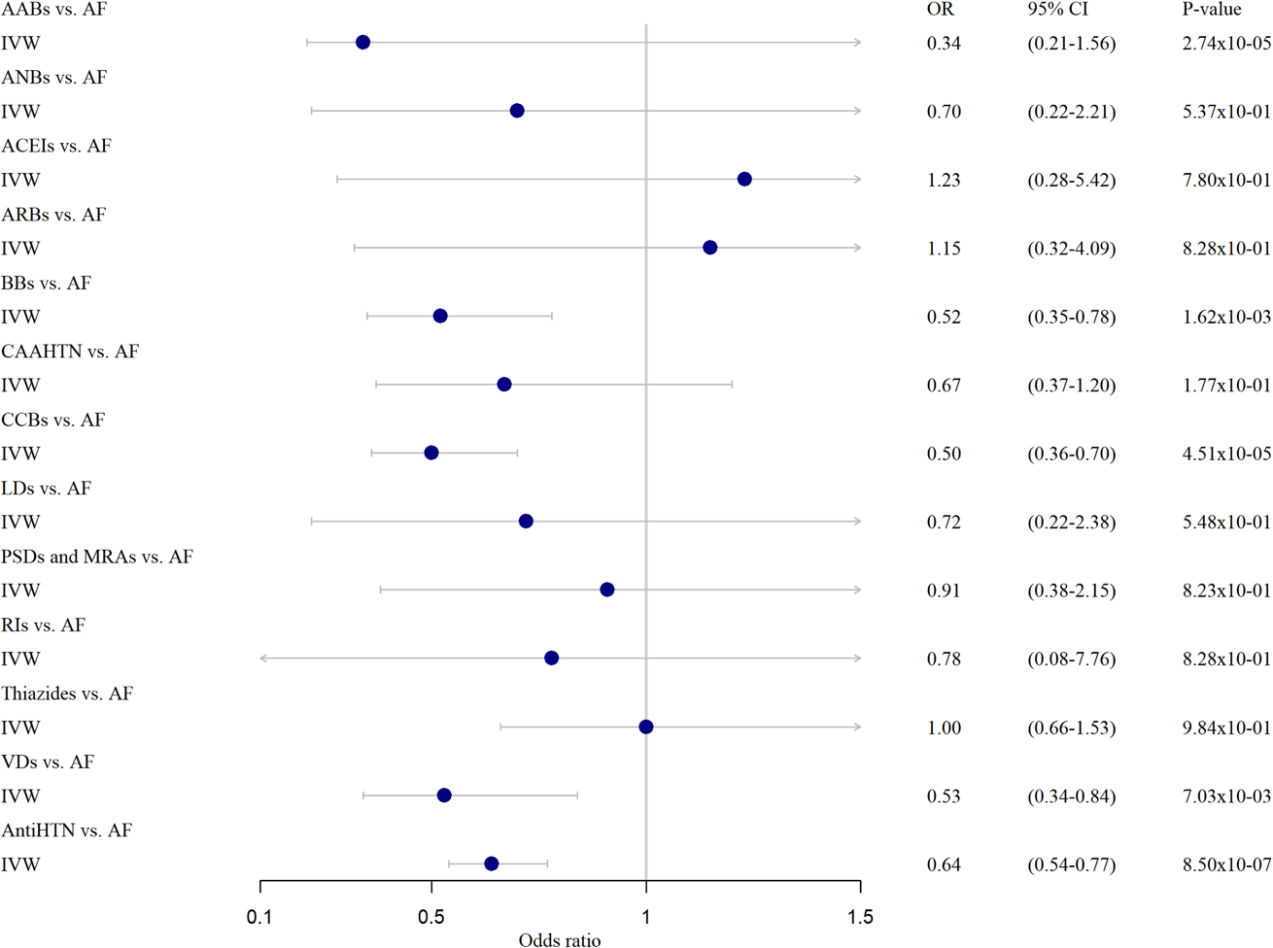
**Forest plot visualizing the Mendelian randomization analyses between antihypertensive drug classes and atrial fibrillation.** Odds ratios represent a genetically determined 10 unit decrease of ln(SBP) through the various antihypertensive drug classes with the odds of atrial fibrillation. AABs indicates adrenergic neuron blockers; ACEIs, angiotensin-converting enzyme inhibitors; AF, atrial fibrillation; ANBs, alpha-adrenocepter blockers; AntiHTN; all 12 antihypertensive drug classes combined; ARBs, angiotensin-II receptor antagonists; BBs, beta-adrenoceptor blockers; CAAHTN, centrally acting antihypertensives; CCBs, calcium channel blockers; IVW, inverse-variance weighted; LDs, loop diuretics; n, number; OR, odds ratio; PSDs, potassium-sparing diuretics and aldosterone antagonists; RIs; renin inhibitors; Thiazides; thiazides and related diuretics; VDs, vasodilators.

All instrumental variables/genetic instruments were considered to have sufficient strength (F statistic >10) to be used in our drug target MR analyses.^20^ Based on the IVW method, our drug target MR analyses supported the causal effects of lowering SBP (per 10 mm Hg) via 4 drug classes: alpha-adrenoceptor blockers (n=11 SNPs; OR, 0.34 [95% CI, 0.21–0.56]; *P*=2.74×10^−05^), beta-adrenoceptor blockers (n=17 SNPs; OR, 0.52 [95% CI, 0.35–0.78]; *P*=1.62×10^−03^), calcium channel blockers (n=49 SNPs; OR, 0.50 [95% CI, 0.36–0.70]; *P*=4.51×10^−05^), vasodilators (n=19 SNPs; OR, 0.53 [95% CI, 0.34–0.84]; *P*=7.03×10^−03^), and all 12 antihypertensive drug classes combined (n=158 SNPs; OR, 0.64 [95% CI, 0.54–0.77]; *P*=8.50×10^−07^) on AF risk (Table; Figure 2). We did not find evidence for causal effects (or a protective effect) from the other 8 antihypertensive drug classes (adrenergic neuron blockers, angiotensin-converting enzyme inhibitors, angiotensin-II receptor antagonists, centrally acting antihypertensives, loop diuretics, potassium-sparing diuretics and aldosterone antagonists, renin inhibitors, and thiazides) on AF risk (Table; Figure 2). When accounting for multiple testing (*P*<0.05/13 [3.85×10^−03^]), alpha-adrenoceptor blockers, beta-adrenoceptor blockers, calcium channel blockers, and all 12 antihypertensive drug classes combined remained statistically significant using the IVW method in the primary analyses. Similar results were observed in our secondary analyses using a different set of genetic instruments, although fewer antihypertensive drug classes were available in this set of genetic instruments. In these analyses, MR also provided evidence of the causal effects of lowering SBP (per 10 mm Hg) via 2 drug classes: beta-adrenoceptor blockers (n=6 SNPs; OR, 0.69 [95% CI, 0.56–0.85]; *P*=3.67×10^−04^) and calcium channel blockers (n=24 SNPs; OR, 0.62 [95% CI, 0.56–0.69]; *P*=1.07×10^−17^) on AF risk (Table S1; Figure S2). Similar results were obtained when accounting for multiple testing (*P*<0.05/13 [3.85×10^−03^]) using the IVW method in the secondary analyses. The effect estimates of the genetic variants associated with antihypertensive drugs and AF that were used in our primary and secondary MR analyses are extensively presented in Tables S6 through S22.

**Table.**
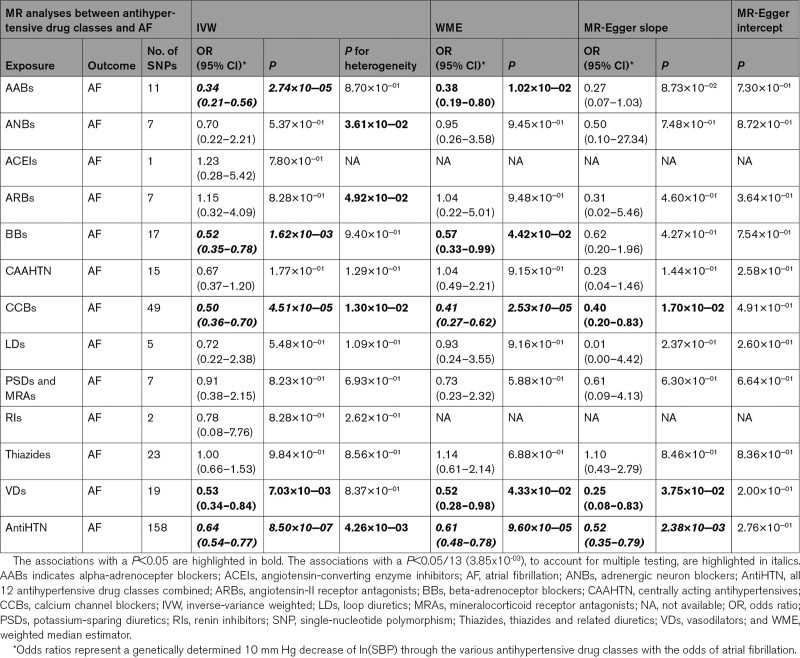
Mendelian Randomization Analyses Between Antihypertensive Drug Classes and Atrial Fibrillation for the Primary Analyses

### MR Sensitivity Analyses

Our MR sensitivity analyses were based on the weighted median estimator (WME) and MR-Egger slope method. The results from these 2 methods were consistent with the results of the IVW method (Table). In detail, the WME method also supported the causal effect of lowering SBP (per 10 mm Hg) via 4 drug classes: alpha-adrenoceptor blockers (n=11 SNPs; OR, 0.38 [95% CI, 0.19–0.80]; *P*=1.02×10^−02^), beta-adrenoceptor blockers (n=17 SNPs; OR, 0.57 [95% CI, 0.33–0.99]; *P*=4.42×10^−02^), calcium channel blockers (n=49 SNPs; OR, 0.41 [95% CI, 0.27–0.62]; *P*=2.53×10^−05^), vasodilators (n=19 SNPs; OR, 0.52 [95% CI, 0.28–0.98]; *P*=4.33×10^−02^), and all 12 antihypertensive drug classes combined (n=158 SNPs; OR, 0.61 [95% CI, 0.48–0.78]; *P*=9.60×10^−05^) on AF risk. When accounting for multiple testing (*P*<0.05/13 [3.85×10^−03^]), only calcium channel blockers and all antihypertensive drug classes combined remained statistically significant using the WME method in the primary analyses. Comparable results were found in our secondary analyses. The WME method also showed a concordant causal effect of lowering SBP per 10 mm Hg via beta-adrenoceptor blockers (n=6 SNPs; OR, 0.69 [95% CI, 0.54–0.89]; *P*=4.45×10^−03^) and calcium channel blockers (n=24 SNPs; OR, 0.62 [95% CI, 0.52–0.73]; *P*=3.74×10^−08^) on AF risk. When accounting for multiple testing (*P*<0.05/13 [3.85×10^−03^]), only calcium channel blockers and all antihypertensive drug classes combined remained statistically significant using the WME method in the secondary analyses. Also, the results of the MR-Egger slope method were consistent with those of the IVW method as well as in the primary as in secondary analyses. After clumping and pruning, the MR-Egger intercept and MR-PRESSO did not provide evidence for the presence of directional pleiotropy (Table; Table S1). We observed similar results when we excluded genetic variants that were also associated with potential confounders and/or horizontal mediators such as myocardial infarction/coronary artery disease,^25^ and heart failure^26^ (Tables S2 and S3).

The exact extent of sample overlap could not be determined due to the unavailability of individual-level data but is based on the individual studies included within the different GWAS. There was potential overlap between SBP and AF for 317 754 individuals in the first study and 77 560 individuals in the second study (Supplemental Methods S1 and S2). We also used an online calculator available at https://sb452.shinyapps.io/overlap that was previously described to estimate the potential impact on our obtained effect estimates caused by sample overlap bias.^17^ Based on the potential overlap as mentioned above (30.8% for the first study and 7.5% for the second study), there was no substantial bias and/or inflation of the type 1 error with a potential sample overlap of 30% (bias 0.032; type 1 error rate 0.10 for the first study and bias 0.006; type 1 error rate 0.05 for the second study).

In addition, we reran our analyses using a more conservative r^2^ for the analyses of all antihypertensive drugs combined, and we found that for the primary analyses, the amount of available SNPs went from 158 to 116 with r^2^ of 0.01 and 73 with r^2^ of 0.001. The effect estimates from the IVW, WME, and MR-Egger slope remained the same and also remained significant (IVW OR, 0.64 [95% CI, 0.54–0.77], *P*=8.50×10^−07^; WME OR, 0.61 [95% CI, 0.48–0.79], *P*=9.60×10^−05^; and MR-Egger slope OR, 0.52 [95% CI, 0.35–0.79], *P*=2.38×10^−03^ in the original analyses versus IVW OR, 0.65 [95% CI, 0.53–0.79], *P*=1.68×10^−05^; WME OR, 0.62 [95% CI, 0.47–0.81], *P*=5.97×10^−04^; and MR-Egger slope OR, 0.47 [95% CI, 0.30–0.75], *P*=2.03×10^−03^ with r^2^ of 0.01 versus IVW OR, 0.60 [95% CI, 0.48–0.75], *P*=4.77×10^−06^; WME OR, 0.59 [95% CI, 0.42–0.82], *P*=1.62×10^−03^; and MR-Egger slope OR, 0.40 [95% CI, 0.24–0.67], *P*=8.36×10^−04^ with r^2^ of 0.001. For the secondary analyses, the number of available SNPs went from 31 to 10 with r^2^ of 0.01 and 8 with r^2^ of 0.001. The effect estimates from the IVW, WME, and MR-Egger slope attenuated, but remained also significant (IVW OR, 0.65 [95% CI, 0.59–0.71], *P*=3.35×10^−19^; WME OR, 0.62 [95% CI, 0.54–0.71], *P*=7.72×10^−12^; and MR-Egger slope OR, 0.68 [95% CI, 0.53–0.88], *P*=6.24×10^−03^ in the original analyses versus IVW OR, 0.96 [95% CI, 0.94–0.98], *P*=7.19×10^−07^; WME OR, 0.95 [95% CI, 0.94–0.97], *P*=9.96×10^−07^; and MR-Egger slope OR, 0.96 [95% CI, 0.92–1.01], *P*=1.45×10^−01^ with r^2^ of 0.01 versus IVW OR, 0.96 [95% CI, 0.94–0.97], *P*=2.29×10^−07^; WME OR, 0.95 [95% CI, 0.93–0.97], *P*=2.76×10^−06^; and MR-Egger slope OR, 0.96 [95% CI, 0.92–1.00], *P*=1.11×10^−01^ with r^2^ of 0.001).

Furthermore, we used FinnGen^28^ as an AF outcome data set to further limit potential sample overlap in our original analyses. In short, the effect estimates were also statistically significant, just as in our original analyses, and the results from these sensitivity analyses also showed a protective effect of antihypertensive drugs on AF. See Tables S4 and S5 for more details.

## DISCUSSION

### Main Findings

This study provides insights into the causal effects of antihypertensive drugs on the risk of AF using a drug target MR framework. Using validated genetic variants, from published studies, that mimic the effect of 12 antihypertensive drug classes, we examined the effect of individual antihypertensive drug classes, as well as their combined effect, on the risk of AF. In this comprehensive MR analysis, we found evidence to support the effect of antihypertensive drugs on AF. More specifically, preventive causal effects on AF risk were observed for alpha-adrenoceptor blockers, beta-adrenoceptor blockers, calcium channel blockers, vasodilators, and all 12 antihypertensive drug classes combined. Our study extends the knowledge regarding the preventive impact of antihypertensive drugs on AF risk.^4,5^ Our findings inform future clinical trials and might have implications for repurposing antihypertensive drugs to prevent AF.

### Interpretation in Light of Existing Evidence

Several mechanisms may explain the complex relationship between hypertension and AF, including structural remodeling, increased ventricular wall tension, and the autonomic nervous system.^1–4,28^ Chronic hypertension may lead to inflammation, fibrosis, and eventually organ damage in the form of atrial myopathy or dilation. Thus, chronic activation of the renin-angiotensin-aldosterone system during hypertension may further exacerbate these pathological processes.^1–4,28^ Also, the hemodynamic effects of hypertension are well known; hypertension increases cardiac afterload, which promotes left ventricular hypertrophy and left ventricular stiffness, decreases ventricular systolic and diastolic function, and increases left atrial pressure. Left atrial dilation is a consequence of this excessive cardiac afterload, which also increases the risk for cardiac dysfunction and AF.^1–4,28^ Hypertension may also dysregulate both the sympathetic and parasympathetic nervous systems, which are both implicated in AF development.^1–4,28^

We observed a possible causal effect of antihypertensive drugs on AF prevention in our study. More specifically, alpha-adrenoceptor blockers, beta-adrenoceptor blockers, calcium channel blockers, vasodilators, and all antihypertensive drug classes combined conferred a reduced AF risk. Regarding the other antihypertensive drug classes, no significant preventive effect was found. Based on our MR results, we hypothesize that drug classes that lower blood pressure through vasodilation rather than through sodium and fluid excretion seem to reduce AF risk. This may suggest that the underlying pathway through which hypertension is targeted may also be of importance, and not only the −10 mm Hg SBP reduction. A previous MR found a causal effect for blood pressure reduction to prevent AF. However, the lack of information on baseline treatment with drugs affecting blood pressure levels was stated as a main limitation, and our study could thereby extend this important study.^29^ This hypothesis seems intuitive given the fact that previous studies have shown differences in the efficacy of various antihypertensive drugs to prevent AF incidence.^4,5^ Albeit we cannot exclude the possibility that we may be underpowered to detect any significant effects for the other antihypertensive drug classes due to a lack of sufficient genetic variants to conduct our analyses. It should be noted that previous studies failed to demonstrate a benefit of beta-adrenoceptor blockers and calcium channel blockers over other drug classes such as angiotensin-converting enzyme inhibitors, angiotensin-II receptor blockers, and diuretics.^30–32^ The potential reduction in AF risk through alpha-adrenoceptor blockers and beta-adrenoceptor blockers is further supported by the ERADICATE-AF atrial, which shows that renal denervation in addition to catheter ablation is beneficial for long-term antiarrhythmic efficacy. This supports the hypothesis that reducing sympathetic activity may lead to an antiarrhythmic effect and fewer reoccurrences of AF in hypertensive AF patients.^33^ Furthermore, we were unable to show a beneficial effect regarding the use of angiotensin-converting enzyme inhibitors and angiotensin-II receptor antagonists to prevent AF, in contrast to prior reports.^4,5^ Although we hypothesized that these drugs would have a protective effect on AF, angiotensin-converting enzyme inhibitors and angiotensin-II receptor blockers may have a positive effect on remodeling of the left atrium and left ventricle, which is known to subsequently reduce the risk of AF. Nevertheless, the absence of evidence does not equal evidence of the absence and increased genetic instruments available for these other drug classes may be required to reconcile these effects. Of note, direct comparison of our results with the used antihypertensive drug formulary in the post hoc analyses from the SPRINT is also challenging, as most participants had a 2- or 3-drug regimen at randomization initiation.^6^ Drug doses were also increased, and/or additional drugs were added during follow-up, if necessary.^6^

It is also worth noting that, when comparing our findings to results from clinical trials or observational studies, there are inherent differences in each methodology. We used genetic proxies to estimate the effect of antihypertensive drugs on AF risk, and thereby our effect estimates represent a lifetime genetic exposure to antihypertensive drugs. In contrast, these other clinical studies estimate the effect of antihypertensive drugs on the patient during a limited duration while using multiple drug classes with different dosages and a defined time point in the life of the patient, for example, a 10-year risk. This may also affect and explain the differences in the magnitude of our obtained effect estimates when one is compare with the magnitude of the treatment effects from clinical trials and/or observational studies. Additionally, unmeasured/residual confounding and reverse causation could still be present in traditional observational studies, which may partly explain some discrepancies. In principle, MR circumvents these biases by leveraging genetic proxies of exposures or risk factors that are less prone to these biases due to the random distribution of genetic variants at conception. Lastly, MR is a reliable research method to examine the causality of associations that are difficult or impractical to study with RCTs due to limitations such as being high-risk, unethical, and/or cost-prohibitive. As one can imagine, it may not be ethical and/or feasible to withhold antihypertensive treatment to patients when conducting a clinical trial.

### Strengths and Limitations

The major strengths of the present study involve the use of validated genetic proxies of antihypertensive drugs and the use of summary statistics from the largest GWAS meta-analyses to date. By using a drug target MR approach, we were able to evaluate the complex interaction between hypertension and AF. Additionally, using an MR approach, we mitigated biases that are more common in traditional observational studies, such as unmeasured/residual confounding and reverse causation. Noteworthy, we used 2 separate exposure data sets and 2 separate outcome data sets to verify our initial findings and to show that our findings were robust. Nonetheless, this study also has some limitations. First, we cannot completely rule out unobserved horizontal pleiotropy. We attempted to identify and correct for horizontal pleiotropy through WME, MR-Egger, MR-PRESSO, and sensitivity plots. In addition, we excluded genetic variants that were associated with potential confounders and/or horizontal mediators such as myocardial infarction/coronary artery disease, and heart failure. Second, there was a potential partial overlap in the study populations that were used to obtain the genetic instruments, which may cause bias towards the observational findings.^17^ However, bias due to sample overlap is difficult to avoid entirely as large-scale genetic consortia need to combine their study samples to optimize statistical power. Also, weak instrument bias is a possibility but remains unlikely, as all included genetic instruments used in our analyses were well validated, as suggested by their reported F statistics.^17^ Furthermore, our study could have been underpowered to detect significant findings for other antihypertensive drug classes due to a lack of sufficient genetic variants for these drug classes. Future GWAS could lead to the identification of a larger number of genetic variants for various antihypertensive drug classes. Lastly, our findings may not be generalizable to younger study populations and other ethnicities because our analyses mainly comprised older individuals of European ancestry. This is noteworthy as it is well established that AF incidence, hypertension incidence, and response to various drug classes vary per ethnicity.

### Perspectives

The ESC guidelines for the diagnosis and management of AF recognize that hypertension is the most common risk factor that is associated with AF development.^1^ Hypertension also further magnifies additional complications, such as stroke and heart failure, and increases the risk of bleeding in AF patients.^1^ Despite this established relationship between hypertension and AF, the evidence regarding the added value of antihypertensive drugs in AF prevention is mostly limited to studies that were retrospective, observational, post hoc analyses, done in selected AF patient populations, or only investigated a specific antihypertensive drug.^4,5^ The SPRINT trial has done promising work and showed that intensive blood pressure treatment reduces the risk of AF in hypertensive patients. Nevertheless, large RCTs and meta-analyses regarding the primary prevention of AF using antihypertensives are lacking thus far. Not surprisingly, RCTs that study (new) drugs are challenging to conduct. Future GWAS could aid in identifying a larger number of SNPs to conduct MR. These MR studies, just as our MR study, could further inform future clinical trials. MR is a helpful tool to aid RCTs or study outcomes that are not feasible to be studied in a clinical setting. Moreover, alternative options to treat hypertension next to antihypertensive medication, such as diet and lifestyle changes, weight loss, smoking cessation, and so on, should remain the cornerstone of primary AF prevention.

### Conclusions

To conclude, by using genetic proxies of antihypertensive drugs, our study confirms that lowering SBP is promising for AF prevention. Our results inform future clinical trials and have implications for repurposing antihypertensive drugs to prevent AF.

## ARTICLE INFORMATION

### Acknowledgments

The authors would like to thank the CHARGE Consortium and individual studies for making their genome-wide association study summary statistics publicly available. S. Geurts, M.J. Tilly, and M. Kavousi conceptualized and wrote the original draft. S. Geurts and M.J. Tilly are responsible for data curation, formal analysis, resource collection, and visualization of the study. M. Kavousi participates in acquisition of funding and supervises the study. S. Geurts, M.J. Tilly, B.H.C. Stricker, J.W. Deckers, N.M.S. de Groot, C.L. Miller, M.A. Ikram, and M. Kavousi are responsible for the investigation of the study. S. Geurts, M.J. Tilly, C.L. Miller, and M. Kavousi wrote the paper methodology. All authors wrote, reviewed, and edited the manuscript.

### Sources of Funding

This study is supported by the Senior Scientist Grant from Dutch Heart Foundation (03-004-2021-T050).

### Disclosures

M.A. Ikram reports consulting fees from BioGen Inc. The other authors report no conflicts.

### Supplemental Material

Supplemental Methods

Figures S1 and S2

Tables S1–S22

## Supplementary Material

**Figure:**