# Supplementary material for: Antihypertensive Drugs for the Prevention of Atrial Fibrillation: A Drug Target Mendelian Randomization Study
Source: Hypertension. 2024 Jun 19;81(8):1766–75. doi: 10.1161/HYPERTENSIONAHA.123.21858 (PMC11251507; doi:10.1161/HYPERTENSIONAHA.123.21858)
Supplement: Supplementary file 1 [file hyp-81-1766-s001.docx]

**Online supplement

Title: Antihypertensive drugs for the prevention of atrial fibrillation: a drug target Mendelian randomization study**Short title: *Antihypertensive drugs and AF: a drug target MR*

Sven Geurts MD, MSc,^1 *^ Martijn J. Tilly MD, MSc,^1 *^ Zuolin Lu MSc,^1^ Bruno H.C. Stricker MD, PhD,^1^ Jaap W. Deckers MD, PhD,^1^ Natasja M.S. de Groot MD, PhD,^2^ Clint L. Miller PhD, ^3^ M. Arfan Ikram MD, PhD^1^, Maryam Kavousi MD, PhD^1^* These authors contributed equally to this work and share first authorship. ^1^ Department of Epidemiology, Erasmus MC, University Medical Center Rotterdam, Rotterdam, The Netherlands
^2^ Department of Cardiology, Erasmus MC, University Medical Center Rotterdam, Rotterdam, The Netherlands
^3^ Department of Biochemistry and Molecular Genetics, University of Virginia, Charlottesville, VA, USA

**Corresponding author:**Dr. M. Kavousi
Department of Epidemiology, Erasmus MC, University Medical Center Rotterdam,
Office Na-2714 PO Box 2040, 3000 CA Rotterdam, The Netherlands
E-mail: [m.kavousi@erasmusmc.nl](mailto:m.kavousi@erasmusmc.nl)

**SUPPLEMENTARY MATERIAL
Methods S1. Study population of the studies from which the genetic instruments for antihypertensive drugs were obtained
Methods S2. Study population of the genome-wide association study from which the genetic instruments for atrial fibrillation were obtained
Methods S3. Mendelian randomization sensitivity analyses
Methods S4. Study population of the genome-wide association study from which the genetic instruments for the potential confounders and/or horizontal mediators were obtained
Table S1. Mendelian randomization analyses between antihypertensive drug classes and atrial fibrillation for the secondary analyses
Table S2. Mendelian randomization analyses between antihypertensive drug classes and atrial fibrillation for the primary analyses without potential confounders and/or horizontal mediators
Table S3. Mendelian randomization analyses between antihypertensive drug classes and atrial fibrillation for the secondary analyses without potential confounders and/or horizontal mediators
Table S4. Mendelian randomization analyses between antihypertensive drug classes and atrial fibrillation using FinnGen data as AF outcome dataset for the primary analyses
Table S5. Mendelian randomization analyses between antihypertensive drug classes and atrial fibrillation using FinnGen data as AF outcome dataset for the secondary analyses
Table S6. Effect estimates for the associations of the genetic variants with alpha-adrenoceptor blockers and atrial fibrillation for the primary analyses
Table S7. Effect estimates for the associations of the genetic variants with adrenergic neuron blockers and atrial fibrillation for the primary analyses
Table S8. Effect estimates for the associations of the genetic variants with angiotensin-converting enzyme inhibitors and atrial fibrillation for the primary analyses
Table S9. Effect estimates for the associations of the genetic variants with angiotensin-II receptor blockers and atrial fibrillation for the primary analyses
Table S10. Effect estimates for the associations of the genetic variants with beta-adrenoceptor blockers and atrial fibrillation for the primary analyses
Table S11. Effect estimates for the associations of the genetic variants with centrally acting antihypertensive drugs and atrial fibrillation for the primary analyses
Table S12. Effect estimates for the associations of the genetic variants with calcium channel blockers and atrial fibrillation for the primary analyses
Table S13. Effect estimates for the associations of the genetic variants with loop diuretics and atrial fibrillation for the primary analyses
Table S14. Effect estimates for the associations of the genetic variants with potassium-sparing diuretics and mineralocorticoid receptor antagonists and atrial fibrillation for the primary analyses
Table S15. Effect estimates for the associations of the genetic variants with renin inhibitors and atrial fibrillation for the primary analyses
Table S16. Effect estimates for the associations of the genetic variants with thiazides and related diuretic agents and atrial fibrillation for the primary analyses
Table S17. Effect estimates for the associations of the genetic variants with vasodilators and atrial fibrillation for the primary analyses
Table S18. Effect estimates for the associations of the genetic variants with all 12 antihypertensive drug classes combined and atrial fibrillation for the primary analyses
Table S19. Effect estimates for the associations of the genetic variants with angiotensin-converting enzyme inhibitors and atrial fibrillation for the secondary analyses
Table S20. Effect estimates for the associations of the genetic variants with beta-adrenoceptor blockers and atrial fibrillation for the secondary analyses
Table S21. Effect estimates for the associations of the genetic variants with calcium channel blockers and atrial fibrillation for the secondary analyses
Table S22. Effect estimates for the associations of the genetic variants with all 3 antihypertensive drug classes combined and atrial fibrillation for the secondary analyses
Figure S1. Flow chart for the selection of genetic variants for the secondary analyses
Figure S2. Forest plot which visualizes the Mendelian randomization analyses between antihypertensive drug classes and atrial fibrillation for the secondary analyses**

**Methods S1. Study population of the studies from which the genetic instruments for antihypertensive drugs were obtained**The first study that we used, identified genetic variants that simulate the action of up to 12 antihypertensive drug classes. Walker et al.(11) used the DrugBank database (<https://go.drugbank.com/>) to identify corresponding target genes and proteins that are modulated by the aforementioned antihypertensive drug classes. Further, Walker et al.(11) used the GTEx database,(13) a database that contains expression quantitative trait loci analyses of 48 tissues from 714 donors, to identify the best genetic variants to instrument each corresponding protein target identified by the DrugBank database. Subsequently, Walker et al.(11) validated the genetic variants by estimating their effect on systolic blood pressure (SBP) by using a two-sample MR analysis. Genetic variants were retained in the following MR analysis if the genetic variants had an effect on SBP. This methodology led to a total of 293 genetic variants that were nominally significantly (p<0.05) associated with SBP. We utilized these published antihypertensive drugs genetic variants, in genes regulating target protein of these drugs, as individual exposures in our primary drug target MR analyses and also as a combined exposure to evaluate its effect on AF.

The GWAS meta-analysis of SBP that Walker et al.(11) used to conduct their two-sample MR to validate their genetic instruments was from the UK Biobank cohort.^13^ This GWAS by Neale et al.(15) encompassed n=317,754 participants from European descent (n=317,754, 100%).

The second study that we used, identified genetic variants that imitate the action of up to 3 antihypertensive drug classes. Gill et al.(12) also used the DrugBank database (<https://go.drugbank.com/>) to identify corresponding target genes and proteins that are influenced by the aforesaid antihypertensive drug classes. Furthermore, Gill et al.(11) used the GeneCards database,(14) a database that contains information regarding the genomic position of the genes, promotors, and/or enhancers of interest, to identify the best genetic variants to instrument each corresponding protein target identified by the DrugBank database. Afterwards, Gill et al.(12) validated the genetic variants by looking up these genetic variants that were genome-wide significantly (p<5x10^-08^) associated with SBP. This led to a total of 31 genetic variants that were genome-wide significantly (p<5x10^-08^) associated with SBP. We again employed these published antihypertensive drugs genetic variants, in genes regulating target protein of these drugs, as individual exposures in our secondary drug target MR analyses and also as a combined exposure to evaluate its effect on AF.

**Methods S1. Study population of the studies from which the genetic instruments for antihypertensive drugs were obtained (continued)**
The GWAS meta-analysis of SBP that Gill et al.(12) used to conduct their two-sample MR to validate their genetic instruments was from the UK Biobank and the International Consortium of Blood Pressure.(16) This GWAS by Evangelou et al.(15) encompassed n=757,601 participants from European descent (n=757,601, 100%).

| Studies or consortium | N of participants | Ethnicity | Phenotype definition |
| --- | --- | --- | --- |
| First study: UK Biobank *   Second study: UK Biobank and the International Consortium of Blood Pressure ** | First study: Discovery analysis in 317,754 individuals.   Second study: Discovery analysis in 757,601 individuals. | First study: European.  Second study: European. | First study: SBP was assessed as a continuous measurement and was log transformed.  Second study: SBP was assessed as a continuous measurement and was log transformed. |
| Abbreviations of the included studies * ** | | | |
| **First study: UK Biobank**.   Second study: **AGES**, **ARIC**, ASCOT-SC, ASCOT-UK, ASPS, B58C, BHS, BRIGHT, **CHS**, Cilento-study, COLAUS, CORO ALL, CROATIA-Korcula, CROATIA-Split, CROATIA-Vis, EGCUT, EGCUT2, EPIC, EPIC-CVD, EPIC-InterAct, EPIC-Norfolk, ERF, Fenland, **FHS**, FINNRISK CASE ALL, FINRISK CTRL ALL, FUSION, GAPP, GoDARTS, GRAPHIC, **GS:SFHS**, GWAS-Fenland, HCS, Health ABC, HTO, H2000 ALL, INGI-VB, INGI-CARL, INGI-FVG, IPM, JUPITER, **KORAS3**, KORAS4, LBC1921, LBC1936, LIFELINES, LOLIPOP EW610, MDC, **MESA**, METSIM, MICROS, MIGen, NESDA, NEO, NSPHS, NTR, OMICS-Fenland, ORCADES, **PIVUS**, **PREVEND**, PROCARDIS, PROSPER, **RS I**, **RS II**, **RS III**, **SHIP**, SardiNIA, STR, TRAILS, TRAILS-CC, TWINSUK, UKHLS, **ULSAM**, **WGHS**, YFS, 3C-DIJON. | | | |

The data with the potential overlapping samples with the SBP GWAS for the first study and second study are marked in **bold**. The exact extent of sample overlap could not be determined due to unavailability of individual level data, but based on the individual studies included within the different GWAS. There was potential overlap between SBP and AF for 317,754 individuals in the first study and 77,560 individuals in the second study.

In addition, we used an online calculator available at <https://sb452.shinyapps.io/overlap> that was previously described(17) to estimate the potential impact on our obtained effect estimates caused by sample overlap bias. Based on the potential overlap as mentioned above (30.8% for the first study and 7.5% for the second study), there was no substantial bias and/or inflation of the type 1 error with a potential sample overlap of 30.8% and 7.5% respectively (bias 0.032; type 1 error rate 0.10 for the first study and bias 0.006; type 1 error rate 0.05 for the second study).

* For further details, please see Neale et al.(15)

** For further details, please see Evangelou et al.(16)

**Methods S2. Study population of the genome-wide association study from which the genetic instruments for atrial fibrillation were obtained**

The AF GWAS meta-analysis examined the binary assessment of AF, and AF cases were defined as individuals with ICD-9: 427.31 or ICD-10: I48. This GWAS by Nielsen et al.(10) encompassed n=1,030,836 participants from mainly European descent (n=1,029,399, 99.9%). The summary statistics of AF were used as an outcome in our drug target MR study.

| Studies or consortium | N of participants | Ethnicity | Phenotype definition |
| --- | --- | --- | --- |
| The Nord-Trøndelag Health Study (HUNT), deCODE, the Michigan Genomics Initiative (MGI), DiscovEHR, UK Biobank, and the AFGen Consortium * ** | Discovery analysis in 1,030,836 individuals.  AF: 60,620 cases, and 970,216 controls. | Mainly European. | AF cases were defined as patients with ICD-9: 427.31 or ICD-10: I48. |
| Abbreviations of the included studies * ** | | | |
| **AGES**, ANGES, **ARIC**, Beat-AF, Biobank Japan, BioMe-Omni, BioMe Omni, BioMe-Omni, BioVU, CCAF, **CHS**, COROGENE, deCODE, DiscovEHR, **FHS**, FINCAVAS, **GS:SFHS**, HUNT, **KORA**, LURIC, MDC-CC/MDCS, **MESA**, MGH AF study, MGH CAMP, MGI, **PIVUS**, **PREVEND**, **PROSPER**, **RS I**, **RS II**, **RS III**, **SHIP**, SPHFC, TwinGene, **UK Biobank**, **ULSAM**, **WGHS**, WTCCC2 Munich. | | | |

The data with the potential overlapping samples with the SBP GWAS for the first study and second study are marked in **bold**. The exact extent of sample overlap could not be determined due to unavailability of individual level data, but based on the individual studies included within the different GWAS. There was potential overlap between SBP and AF for 317,754 individuals in the first study and 77,560 individuals in the second study.

* For further details, please see Nielsen et al.(10)

** For further details, please see Christophersen et al.(18)

**Methods S3. Mendelian randomization sensitivity analyses**

Multiple sensitivity analyses were conducted to obtain reliable and valid causal estimates. First, the MR estimates could be biased if genetic variants have horizontal pleiotropic effects that affect the outcome of interest via other pathways than through the exposure of interest. In an attempt to satisfy the second and third MR assumption, additional analyses were conducted including the weighted median estimator (WME), MR‐Egger and Mendelian Randomization Pleiotropy RESidual Sum and Outlier (MR-PRESSO) to account and formally test for horizontal pleiotropy.(22-24) More specifically, the WME method gives a weighted median effect of genetically determined antihypertensive drugs on AF risk,(24) and this method assumes that only half of the genetic variants needs to be valid genetic instruments (in other words, no violation of the three MR assumptions for half of the genetic variants). Furthermore, if horizontal pleiotropy is absent, the MR-Egger intercept will not significantly deviate from zero and the MR-Egger slope will be in line with the IVW and WME methods.(23) To put it another way, similar effect estimates from the IVW, WME, MR-Egger slope indicate that the MR results are robust.(22-24) Moreover, we used MR-PRESSO and examined the sensitivity plots such as the scatter plots to identify and remove horizontal pleiotropic outliers to provide an outlier-corrected estimate.^26^ Cochran’s Q test was used to test for heterogeneity between genetic variants. Third, we excluded genetic variants that were also associated with potential confounders and/or horizontal mediators of the exposure-outcome association (myocardial infarction/coronary artery disease,(25) and heart failure(26)), since this may bias our estimates. Finally, we investigated the potential overlap between the study samples that were used to identify the genetic variants, because sample overlap in two-sample MR analyses might potentially cause bias towards the observational findings.(17)

**Methods S4. Study population of the genome-wide association study from which the genetic instruments for the potential confounders and/or horizontal mediators were obtained**

| Studies or consortium | N of participants | Ethnicity | Phenotype definition |
| --- | --- | --- | --- |
| UK Biobank and CARDIoGRAMplusC4D * | Discovery analysis in ~638,000 individuals.  MI/CAD: ~61,000 cases, and ~577,000 controls. | Mainly European. | UK Biobank: MI was defined as: 1) Doctor-diagnosed MI; this field is a summary of the distinct primary diagnoses codes a participant has had recorded across all his/her hospital episodes. 2) International Classification of Diseases version-10 (ICD10) primary and secondary. 3) Self-reported MI.  CARDIoGRAMplusC4D: Individual study-specific defined MI. |
| HERMES Consortium ** | Discovery analysis in 977,323 individuals.  HF: 47,309 cases, and 930,014 controls. | European. | HF was defined as a clinical diagnosis of HF of any aetiology with no inclusion criteria based on left ventricular ejection fraction. |

**Abbreviations:** CAD, coronary artery disease; HF, heart failure; MI, myocardial infarction.

* For further details, please see Supplementary Table 1 of the GWAS from Hartiala et al.(25)

** For further details, please see Supplementary Data 12 of the GWAS from Shah et al.(26)

**Table S1. Mendelian randomization analyses between antihypertensive drug classes and atrial fibrillation for the secondary analyses**

|  | | | **IVW** | | | **WME** | | **MR-Egger**  **slope** | | **MR-Egger**  **intercept** |
| --- | --- | --- | --- | --- | --- | --- | --- | --- | --- | --- |
| **Exposure** | **Outcome** | **n of SNPs** | **OR (95% CI) *** | **p** | **p for heterogeneity** | **OR (95% CI) *** | **p** | **OR (95% CI) *** | **p** | **p** |
| **ACEIs** | **AF** | 1 | 1.12 (0.70-1.78) | 6.48x10^-01^ | NA | NA | NA | NA | NA | NA |
| **BBs** | **AF** | 6 | ***0.69 (0.56-0.85)*** | ***3.67x10^-04^*** | 9.17x10^-01^ | **0.69 (0.54-0.89)** | **4.45x10^-03^** | 0.66 (0.33-1.31) | 2.97x10^-01^ | 8.90x10^-01^ |
| **CCBs** | **AF** | 24 | ***0.62 (0.56-0.69)*** | ***1.07x10^-17^*** | 7.10x10^-01^ | ***0.62 (0.52-0.73)*** | ***3.74x10^-08^*** | **0.68 (0.52-0.90)** | **1.25x10^-02^** | 4.67x10^-01^ |
| **AntiHTN** | **AF** | 31 | ***0.65 (0.59-0.71)*** | ***3.35x10^-19^*** | 6.56x10^-01^ | ***0.62 (0.54-0.71)*** | ***7.72x10^-12^*** | **0.68 (0.53-0.88)** | **6.24x10^-03^** | 6.70x10^-01^ |

**Abbreviations:** ACEIs, angiotensin-converting enzyme inhibitors; AF, atrial fibrillation; AntiHTN; all 3 antihypertensive drug classes combined; CCBs, calcium channel blockers; CI, confidence interval; IVW, inverse variance weighted; n, number; OR, odds ratio; SNP, single nucleotide polymorphism; WME, weighted median estimator.

* Odds ratios represent a genetically determined 10 unit decrease of ln(SBP) through the various antihypertensive drug classes with the odds of atrial fibrillation.

The associations with a p-value <0.05 are highlighted in **bold**.
The associations with a p<0.05/13 (3.85x10^-03^) to account for multiple testing are highlighted in *italics*.

**Table S2. Mendelian randomization analyses between antihypertensive drug classes and atrial fibrillation for the primary analyses without potential confounders and/or horizontal mediators**

|  | | | **IVW** | | | **WME** | | **MR-Egger**  **slope** | | **MR-Egger**  **intercept** |
| --- | --- | --- | --- | --- | --- | --- | --- | --- | --- | --- |
| **Exposure** | **Outcome** | **n of SNPs** | **OR (95% CI) *** | **p** | **p for heterogeneity** | **OR (95% CI) *** | **p** | **OR (95% CI) *** | **p** | **p** |
| **AABs** | **AF** | 11 | ***0.34 (0.21-0.56)*** | ***2.74x10^-05^*** | 8.70x10^-01^ | **0.38 (0.19-0.80)** | **1.02x10^-02^** | 0.27 (0.07-1.03) | 8.73x10^-02^ | 7.30x10^-01^ |
| **ANBs** | **AF** | 7 | 0.70 (0.22-2.21) | 5.37x10^-01^ | **3.61x10^-02^** | 0.95 (0.26-3.58) | 9.45x10^-01^ | 0.50 (0.10-27.34) | 7.48x10^-01^ | 8.72x10^-01^ |
| **ACEIs** | **AF** | 1 | 1.23 (0.28-5.42) | 7.80x10^-01^ | NA | NA | NA | NA | NA | NA |
| **ARBs** | **AF** | 7 | 1.15 (0.32-4.09) | 8.28x10^-01^ | **4.92x10^-02^** | 1.04 (0.22-5.01) | 9.48x10^-01^ | 0.31 (0.02-5.46) | 4.60x10^-01^ | 3.64x10^-01^ |
| **BBs** | **AF** | 17 | ***0.52 (0.35-0.78)*** | ***1.62x10^-03^*** | 9.40x10^-01^ | **0.57 (0.33-0.99)** | **4.42x10^-02^** | 0.62 (0.20-1.96) | 4.27x10^-01^ | 7.54x10^-01^ |
| **CAAHTN** | **AF** | 15 | 0.67 (0.37-1.20) | 1.77x10^-01^ | 1.29x10^-01^ | 1.04 (0.49-2.21) | 9.15x10^-01^ | 0.23 (0.04-1.46) | 1.44x10^-01^ | 2.58x10^-01^ |
| **CCBs** | **AF** | 49 | ***0.50 (0.36-0.70)*** | ***4.51x10^-05^*** | **1.30x10^-02^** | ***0.41 (0.27-0.62)*** | ***2.53x10^-05^*** | **0.40 (0.20-0.83)** | **1.70x10^-02^** | 4.91x10^-01^ |
| **LDs** | **AF** | 5 | 0.72 (0.22-2.38) | 5.48x10^-01^ | 1.09x10^-01^ | 0.93 (0.24-3.55) | 9.16x10^-01^ | 0.01 (0.00-4.42) | 2.37x10^-01^ | 2.60x10^-01^ |
| **PSDs and MRAs** | **AF** | 7 | 0.91 (0.38-2.15) | 8.23x10^-01^ | 6.93x10^-01^ | 0.73 (0.23-2.32) | 5.88x10^-01^ | 0.61 (0.09-4.13) | 6.30x10^-01^ | 6.64x10^-01^ |
| **RIs** | **AF** | 2 | 0.78 (0.08-7.76) | 8.28x10^-01^ | 2.62x10^-01^ | NA | NA | NA | NA | NA |
| **Thiazides** | **AF** | 23 | 1.00 (0.66-1.53) | 9.84x10^-01^ | 8.56x10^-01^ | 1.14 (0.61-2.14) | 6.88x10^-01^ | 1.10 (0.43-2.79) | 8.46x10^-01^ | 8.36x10^-01^ |
| **VDs** | **AF** | 18 | 0.58 (0.36-0.94) | 2.59x10^-02^ | 8.48x10^-01^ | 0.55 (0.27-1.09) | 8.69x10^-02^ | 0.29 (0.08-1.03) | 7.35x10^-02^ | 2.67x10^-01^ |
| **AntiHTN** | **AF** | 157 | ***0.65 (0.55-0.78)*** | ***1.72x10^-06^*** | **4.49x10^-03^** | ***0.63 (0.49-0.80)*** | ***2.04x10^-04^*** | ***0.53 (0.35-0.80)*** | ***3.12x10^-03^*** | 2.92x10^-01^ |

**Abbreviations:** AABs, alpha-adrenocepter blockers; ACEIs, angiotensin-converting enzyme inhibitors; AF, atrial fibrillation; ANBs, adrenergic neuron blockers; AntiHTN; all 12 antihypertensive drug classes combined; ARBs, angiotensin-II receptor antagonists; BBs, beta-adrenoceptor blockers; CAAHTN, centrally acting antihypertensives; CCBs, calcium channel blockers; CI, confidence interval; IVW, inverse variance weighted; LDs, loop diuretics; MRAs; mineralocorticoid receptor antagonists; n, number; OR, odds ratio; PSDs, potassium sparing diuretics; RIs; renin inhibitors; SNP, single nucleotide polymorphism; Thiazides; thiazides and related diuretics; VDs, vasodilators; WME, weighted median estimator.

* Odds ratios represent a genetically determined 10 mmHg decrease of ln(SBP) through the various antihypertensive drug classes with the odds of atrial fibrillation.

The associations with a p<0.05 are highlighted in **bold**.
The associations with a p<0.05/13 (3.85x10^-03^) to account for multiple testing are highlighted in *italics*.

**Table S3. Mendelian randomization analyses between antihypertensive drug classes and atrial fibrillation for the secondary analyses without potential confounders and/or horizontal mediators**

|  | | | **IVW** | | | **WME** | | **MR-Egger**  **slope** | | **MR-Egger**  **intercept** |
| --- | --- | --- | --- | --- | --- | --- | --- | --- | --- | --- |
| **Exposure** | **Outcome** | **n of SNPs** | **OR (95% CI) *** | **p** | **p for heterogeneity** | **OR (95% CI) *** | **p** | **OR (95% CI) *** | **p** | **p** |
| **ACEIs** | **AF** | 1 | 1.12 (0.70-1.78) | 6.48x10^-01^ | NA | NA | NA | NA | NA | NA |
| **BBs** | **AF** | 6 | ***0.69 (0.56-0.85)*** | ***3.67x10^-04^*** | 9.17x10^-01^ | **0.69 (0.54-0.89)** | **4.45x10^-03^** | 0.66 (0.33-1.31) | 2.97x10^-01^ | 8.90x10^-01^ |
| **CCBs** | **AF** | 24 | ***0.62 (0.56-0.69)*** | ***1.07x10^-17^*** | 7.10x10^-01^ | ***0.62 (0.52-0.73)*** | ***3.74x10^-08^*** | **0.68 (0.52-0.90)** | **1.25x10^-02^** | 4.67x10^-01^ |
| **AntiHTN** | **AF** | 31 | ***0.65 (0.59-0.71)*** | ***3.35x10^-19^*** | 6.56x10^-01^ | ***0.62 (0.54-0.71)*** | ***7.72x10^-12^*** | **0.68 (0.53-0.88)** | **6.24x10^-03^** | 6.70x10^-01^ |

**Abbreviations:** ACEIs, angiotensin-converting enzyme inhibitors; AF, atrial fibrillation; AntiHTN; all 3 antihypertensive drug classes combined; CCBs, calcium channel blockers; CI, confidence interval; IVW, inverse variance weighted; n, number; OR, odds ratio; SNP, single nucleotide polymorphism; WME, weighted median estimator.

* Odds ratios represent a genetically determined 10 unit decrease of ln(SBP) through the various antihypertensive drug classes with the odds of atrial fibrillation.

The associations with a p-value <0.05 are highlighted in **bold**.
The associations with a p<0.05/13 (3.85x10^-03^) to account for multiple testing are highlighted in *italics*.

**Table S4. Mendelian randomization analyses between antihypertensive drug classes and atrial fibrillation using using FinnGen data as AF outcome dataset for the primary analyses**

|  | | | **IVW** | | | **WME** | | **MR-Egger**  **slope** | | **MR-Egger**  **intercept** |
| --- | --- | --- | --- | --- | --- | --- | --- | --- | --- | --- |
| **Exposure** | **Outcome** | **n of SNPs** | **OR (95% CI) *** | **p** | **p for heterogeneity** | **OR (95% CI) *** | **p** | **OR (95% CI) *** | **p** | **p** |
| **AntiHTN** | **AF** | 155 | **0.35 (0.24-0.51)** | **1.89x10^-08^** | **3.55x10^-02^** | **0.43 (0.26-0.73)** | **1.57x10^-03^** | **0.30 (0.13-0.69)** | **5.71x10^-03^** | 6.66x10^-01^ |

**Abbreviations:** AF, atrial fibrillation; AntiHTN; all 12 antihypertensive drug classes combined; CI, confidence interval; IVW, inverse variance weighted; n, number; OR, odds ratio; SNP, single nucleotide polymorphism; WME, weighted median estimator.

* Odds ratios represent a genetically determined 10 unit decrease of ln(SBP) through the various antihypertensive drug classes with the odds of atrial fibrillation.

The associations with a p-value <0.05 are highlighted in **bold**.

**Table S5. Mendelian randomization analyses between antihypertensive drug classes and atrial fibrillation using FinnGen data as AF outcome dataset for the secondary analyses**

|  | | | **IVW** | | | **WME** | | **MR-Egger**  **slope** | | **MR-Egger**  **intercept** |
| --- | --- | --- | --- | --- | --- | --- | --- | --- | --- | --- |
| **Exposure** | **Outcome** | **n of SNPs** | **OR (95% CI) *** | **p** | **p for heterogeneity** | **OR (95% CI) *** | **p** | **OR (95% CI) *** | **p** | **p** |
| **AntiHTN** | **AF** | 29 | **0.94 (0.91-0.97)** | **1.11x10^-04^** | **6.03 x10^-04^** | **0.93 (0.89-0.97)** | **1.93x10^-04^** | 0.96 (0.88-1.05) | 3.52x10^-01^ | 5.98x10^-01^ |

**Abbreviations:** AF, atrial fibrillation; AntiHTN; all 12 antihypertensive drug classes combined; CI, confidence interval; IVW, inverse variance weighted; n, number; OR, odds ratio; SNP, single nucleotide polymorphism; WME, weighted median estimator.

* Odds ratios represent a genetically determined 10 unit decrease of ln(SBP) through the various antihypertensive drug classes with the odds of atrial fibrillation.

The associations with a p-value <0.05 are highlighted in **bold**.

**Table S6. Effect estimates for the associations of the genetic variants with alpha-adrenoceptor blockers and atrial fibrillation for the primary analyses**

|  | | | | **Exposure effect estimates** | | | | | | | | | **Outcome effect estimates** | | | |
| --- | --- | --- | --- | --- | --- | --- | --- | --- | --- | --- | --- | --- | --- | --- | --- | --- |
| **SNP** | **Chr** | **Pos** | **Gene** | **Trait** | **EA** | **OA** | **EAF** | **Beta** | **SE** | **Pval** | **Trait** | **EAF** | | **Beta** | **SE** | **Pval** |
| rs17645325 | 5 | 158476709 | ADRA1B | AABs | C | T | 0.1251 | 0.014443 | 0.003713 | 1.00E-04 | AF | 0.1268 | | 0.006600 | 0.010200 | 5.18E-01 |
| rs143589638 | 5 | 159629575 | ADRA1B | AABs | G | A | 0.0520 | -0.011825 | 0.005550 | 3.31E-02 | AF | 0.0486 | | -0.011200 | 0.015300 | 4.66E-01 |
| rs77185818 | 8 | 26288222 | ADRA1A | AABs | A | G | 0.1200 | -0.008523 | 0.003794 | 2.47E-02 | AF | 0.1194 | | -0.001800 | 0.010700 | 8.63E-01 |
| rs1899494 | 8 | 26398643 | ADRA1A | AABs | A | C | 0.2605 | 0.010537 | 0.002801 | 1.69E-04 | AF | 0.2745 | | 0.004600 | 0.007400 | 5.32E-01 |
| rs12416245 | 10 | 111938114 | ADRA2A | AABs | G | T | 0.1873 | 0.008922 | 0.003152 | 4.64E-03 | AF | 0.1827 | | 0.020600 | 0.008600 | 1.66E-02 |
| rs6585052 | 10 | 113046403 | ADRA2A | AABs | T | C | 0.9879 | 0.026214 | 0.011265 | 2.00E-02 | AF | 0.9516 | | 0.034000 | 0.026900 | 2.06E-01 |
| rs4366419 | 10 | 113049557 | ADRA2A | AABs | G | A | 0.9188 | 0.012867 | 0.004513 | 4.36E-03 | AF | 0.8939 | | 0.019600 | 0.011900 | 1.01E-01 |
| rs4072884 | 11 | 1482720 | TH | AABs | T | C | 0.5900 | -0.005547 | 0.002513 | 2.73E-02 | AF | 0.5906 | | -0.002800 | 0.006900 | 6.84E-01 |
| rs71455916 | 11 | 2005794 | TH | AABs | A | C | 0.1938 | 0.018705 | 0.003124 | 2.12E-09 | AF | 0.2037 | | 0.026800 | 0.009000 | 2.96E-03 |
| rs3842756 | 11 | 2180772 | TH | AABs | T | C | 0.2274 | -0.006694 | 0.002935 | 2.26E-02 | AF | 0.2237 | | -0.011800 | 0.008200 | 1.52E-01 |
| rs55778060 | 20 | 4189771 | ADRA1D | AABs | T | C | 0.1250 | 0.010754 | 0.003810 | 4.76E-03 | AF | 0.1223 | | 0.006500 | 0.010700 | 5.45E-01 |

**Abbreviations:** AABs, alpha-adrenocepter blockers; AF, atrial fibrillation; Chr, chromosome; EA, effect allele; EAF, effect allele frequency; OA, other allele; Pos, genomic position; Pval, p-value; SE, standard error; SNP, single nucleotide polymorphism.

**Table S7. Effect estimates for the associations of the genetic variants with adrenergic neuron blockers and atrial fibrillation for the primary analyses**

|  | | | | **Exposure effect estimates** | | | | | | | | | **Outcome effect estimates** | | | |
| --- | --- | --- | --- | --- | --- | --- | --- | --- | --- | --- | --- | --- | --- | --- | --- | --- |
| **SNP** | **Chr** | **Pos** | **Gene** | **Trait** | **EA** | **OA** | **EAF** | **Beta** | **SE** | **Pval** | **Trait** | **EAF** | | **Beta** | **SE** | **Pval** |
| rs62153010 | 2 | 96854568 | ADRA2B | ANBs | A | G | 0.1699 | -0.010002 | 0.003277 | 2.27E-03 | AF | 0.1697 | | 0.009900 | 0.008900 | 2.66E-01 |
| rs12416245 | 10 | 111938114 | ADRA2A | ANBs | G | T | 0.1873 | 0.008922 | 0.003152 | 4.64E-03 | AF | 0.1827 | | 0.020600 | 0.008600 | 1.66E-02 |
| rs76100968 | 10 | 112887951 | ADRA2A | ANBs | T | C | 0.0628 | 0.010218 | 0.005060 | 4.35E-02 | AF | 0.0656 | | -0.004600 | 0.015300 | 7.62E-01 |
| rs6585052 | 10 | 113046403 | ADRA2A | ANBs | T | C | 0.9879 | 0.026214 | 0.011265 | 2.00E-02 | AF | 0.9516 | | 0.034000 | 0.026900 | 2.06E-01 |
| rs4366419 | 10 | 113049557 | ADRA2A | ANBs | G | A | 0.9188 | 0.012867 | 0.004513 | 4.36E-03 | AF | 0.8939 | | 0.019600 | 0.011900 | 1.01E-01 |
| rs4918689 | 10 | 113518004 | ADRA2A | ANBs | C | A | 0.0706 | 0.012865 | 0.004822 | 7.63E-03 | AF | 0.0746 | | -0.022000 | 0.012800 | 8.47E-02 |
| rs73569213 | 11 | 128478380 | KCNJ1 | ANBs | A | G | 0.1779 | -0.006528 | 0.003213 | 4.22E-02 | AF | 0.1792 | | -0.000400 | 0.008800 | 9.64E-01 |

**Abbreviations:** AF, atrial fibrillation; ANBs, adrenergic neuron blockers; Chr, chromosome; EA, effect allele; EAF, effect allele frequency; OA, other allele; Pos, genomic position; Pval, p-value; SE, standard error; SNP, single nucleotide polymorphism.

**Table S8. Effect estimates for the associations of the genetic variants with angiotensin-converting enzyme inhibitors and atrial fibrillation for the primary analyses**

|  | | | | **Exposure effect estimates** | | | | | | | | | **Outcome effect estimates** | | | |
| --- | --- | --- | --- | --- | --- | --- | --- | --- | --- | --- | --- | --- | --- | --- | --- | --- |
| **SNP** | **Chr** | **Pos** | **Gene** | **Trait** | **EA** | **OA** | **EAF** | **Beta** | **SE** | **Pval** | **Trait** | **EAF** | | **Beta** | **SE** | **Pval** |
| rs4968783 | 17 | 61550729 | ACE | ACEIs | A | C | 0.6194 | -0.009012 | 0.002544 | 3.97E-04 | AF | 0.5955 | | 0.001900 | 0.006800 | 7.84E-01 |

**Abbreviations:** ACEIs, angiotensin-converting enzyme inhibitors; alpha-adrenocepter blockers; AF, atrial fibrillation; Chr, chromosome; EA, effect allele; EAF, effect allele frequency; OA, other allele; Pos, genomic position; Pval, p-value; SE, standard error; SNP, single nucleotide polymorphism.

**Table S9. Effect estimates for the associations of the genetic variants with angiotensin-II receptor blockers and atrial fibrillation for the primary analyses**

|  | | | | **Exposure effect estimates** | | | | | | | | | **Outcome effect estimates** | | | |
| --- | --- | --- | --- | --- | --- | --- | --- | --- | --- | --- | --- | --- | --- | --- | --- | --- |
| **SNP** | **Chr** | **Pos** | **Gene** | **Trait** | **EA** | **OA** | **EAF** | **Beta** | **SE** | **Pval** | **Trait** | **EAF** | | **Beta** | **SE** | **Pval** |
| rs9829399 | 3 | 11652673 | PPARG | ARBs | T | C | 0.1172 | 0.008104 | 0.003822 | 3.40E-02 | AF | 0.1211 | | 0.000400 | 0.010000 | 9.65E-01 |
| rs12489143 | 3 | 148105556 | AGTR1 | ARBs | G | A | 0.0425 | 0.012518 | 0.006082 | 3.96E-02 | AF | 0.0434 | | -0.006200 | 0.015400 | 6.89E-01 |
| rs59252002 | 3 | 148137212 | AGTR1 | ARBs | C | T | 0.0976 | 0.008680 | 0.004141 | 3.61E-02 | AF | 0.0984 | | -0.020600 | 0.011100 | 6.39E-02 |
| rs80350379 | 3 | 148485148 | AGTR1 | ARBs | T | C | 0.0167 | -0.021258 | 0.009573 | 2.64E-02 | AF | 0.0293 | | 0.041100 | 0.025300 | 1.05E-01 |
| rs73015088 | 3 | 148832218 | AGTR1 | ARBs | T | C | 0.0076 | -0.030585 | 0.014234 | 3.17E-02 | AF | 0.0068 | | -0.030600 | 0.038300 | 4.25E-01 |
| rs118123032 | 3 | 148913426 | AGTR1 | ARBs | T | C | 0.0328 | -0.022378 | 0.006917 | 1.22E-03 | AF | 0.0355 | | -0.040600 | 0.019100 | 3.35E-02 |
| rs79387447 | 3 | 149370293 | AGTR1 | ARBs | A | G | 0.0134 | -0.021610 | 0.010723 | 4.39E-02 | AF | 0.0130 | | 0.039700 | 0.034400 | 2.49E-01 |

**Abbreviations:** AF, atrial fibrillation; ARBs, angiotensin-II receptor antagonists; Chr, chromosome; EA, effect allele; EAF, effect allele frequency; OA, other allele; Pos, genomic position; Pval, p-value; SE, standard error; SNP, single nucleotide polymorphism.

**Table S10. Effect estimates for the associations of the genetic variants with beta-adrenoceptor blockers and atrial fibrillation for the primary analyses**

|  | | | | **Exposure effect estimates** | | | | | | | | | **Outcome effect estimates** | | | |
| --- | --- | --- | --- | --- | --- | --- | --- | --- | --- | --- | --- | --- | --- | --- | --- | --- |
| **SNP** | **Chr** | **Pos** | **Gene** | **Trait** | **EA** | **OA** | **EAF** | **Beta** | **SE** | **Pval** | **Trait** | **EAF** | | **Beta** | **SE** | **Pval** |
| rs115412659 | 5 | 148150080 | ADRB2 | BBs | A | G | 0.0582 | 0.012452 | 0.005290 | 1.86E-02 | AF | 0.0507 | | 0.034200 | 0.017000 | 4.35E-02 |
| rs10067003 | 5 | 148547490 | ADRB2 | BBs | T | C | 0.2422 | 0.008417 | 0.002905 | 3.77E-03 | AF | 0.2415 | | -0.005100 | 0.007900 | 5.22E-01 |
| rs11750184 | 5 | 148846518 | ADRB2 | BBs | A | G | 0.2665 | -0.006937 | 0.002791 | 1.30E-02 | AF | 0.2615 | | -0.005300 | 0.007500 | 4.80E-01 |
| rs17645325 | 5 | 158476709 | ADRA1B | BBs | C | T | 0.1251 | 0.014443 | 0.003713 | 1.00E-04 | AF | 0.1268 | | 0.006600 | 0.010200 | 5.18E-01 |
| rs12654978 | 5 | 159006314 | ADRA1B | BBs | T | C | 0.3464 | -0.005126 | 0.002594 | 4.82E-02 | AF | 0.3260 | | -0.000600 | 0.007200 | 9.35E-01 |
| rs143589638 | 5 | 159629575 | ADRA1B | BBs | G | A | 0.0520 | -0.011825 | 0.005550 | 3.31E-02 | AF | 0.0486 | | -0.011200 | 0.015300 | 4.66E-01 |
| rs144549900 | 7 | 150244771 | KCNH2 | BBs | C | A | 0.0662 | -0.012999 | 0.005023 | 9.66E-03 | AF | 0.0734 | | -0.022800 | 0.014200 | 1.10E-01 |
| rs77185818 | 8 | 26288222 | ADRA1A | BBs | A | G | 0.1200 | -0.008523 | 0.003794 | 2.47E-02 | AF | 0.1194 | | -0.001800 | 0.010700 | 8.63E-01 |
| rs1899494 | 8 | 26398643 | ADRA1A | BBs | A | C | 0.2605 | 0.010537 | 0.002801 | 1.69E-04 | AF | 0.2745 | | 0.004600 | 0.007400 | 5.32E-01 |
| rs35866749 | 8 | 38070128 | ADRB3 | BBs | G | A | 0.1547 | -0.010636 | 0.003403 | 1.77E-03 | AF | 0.1485 | | -0.008300 | 0.010400 | 4.28E-01 |
| rs151591 | 10 | 115713566 | ADRB1 | BBs | G | A | 0.7674 | 0.012450 | 0.002918 | 1.98E-05 | AF | 0.7555 | | 0.007600 | 0.007900 | 3.34E-01 |
| rs74717224 | 10 | 115719872 | ADRB1 | BBs | T | G | 0.0586 | -0.014011 | 0.005245 | 7.55E-03 | AF | 0.0573 | | -0.005700 | 0.014400 | 6.93E-01 |
| rs11196589 | 10 | 115779717 | ADRB1 | BBs | C | A | 0.4039 | 0.005045 | 0.002531 | 4.62E-02 | AF | 0.3995 | | 0.011600 | 0.006900 | 9.09E-02 |
| rs7076938 | 10 | 115789375 | ADRB1 | BBs | T | C | 0.7350 | 0.017966 | 0.002794 | 1.28E-10 | AF | 0.7287 | | 0.010300 | 0.007500 | 1.71E-01 |
| rs7093444 | 10 | 115794324 | ADRB1 | BBs | C | T | 0.1275 | -0.009607 | 0.003694 | 9.31E-03 | AF | 0.1368 | | -0.015000 | 0.009600 | 1.18E-01 |
| rs735710 | 20 | 3692839 | ADRA1D | BBs | C | T | 0.0704 | -0.014293 | 0.004855 | 3.24E-03 | AF | 0.0727 | | -0.003200 | 0.013000 | 8.07E-01 |
| rs55778060 | 20 | 4189771 | ADRA1D | BBs | T | C | 0.1250 | 0.010754 | 0.003810 | 4.76E-03 | AF | 0.1223 | | 0.006500 | 0.010700 | 5.45E-01 |

**Abbreviations:** AF, atrial fibrillation; BBs, beta-adrenoceptor blockers; Chr, chromosome; EA, effect allele; EAF, effect allele frequency; OA, other allele; Pos, genomic position; Pval, p-value; SE, standard error; SNP, single nucleotide polymorphism.

**Table S11. Effect estimates for the associations of the genetic variants with centrally acting antihypertensive drugs and atrial fibrillation for the primary analyses**

|  | | | | **Exposure effect estimates** | | | | | | | | | **Outcome effect estimates** | | | |
| --- | --- | --- | --- | --- | --- | --- | --- | --- | --- | --- | --- | --- | --- | --- | --- | --- |
| **SNP** | **Chr** | **Pos** | **Gene** | **Trait** | **EA** | **OA** | **EAF** | **Beta** | **SE** | **Pval** | **Trait** | **EAF** | | **Beta** | **SE** | **Pval** |
| rs62153010 | 2 | 96854568 | ADRA2B | CAAHTN | A | G | 0.1699 | -0.010002 | 0.003277 | 2.27E-03 | AF | 0.1697 | | 0.009900 | 0.008900 | 2.66E-01 |
| rs524986 | 3 | 52228496 | NISCH | CAAHTN | C | T | 0.5333 | 0.007881 | 0.002466 | 1.39E-03 | AF | 0.5444 | | -0.000200 | 0.006700 | 9.74E-01 |
| rs149028979 | 3 | 53132630 | NISCH | CAAHTN | A | G | 0.0460 | 0.023519 | 0.005998 | 8.82E-05 | AF | 0.0436 | | 0.035300 | 0.017700 | 4.63E-02 |
| rs7610701 | 3 | 53176264 | NISCH | CAAHTN | G | T | 0.5785 | 0.007200 | 0.002693 | 7.51E-03 | AF | 0.5836 | | -0.006600 | 0.007600 | 3.84E-01 |
| rs6764111 | 3 | 53207401 | NISCH | CAAHTN | G | A | 0.7725 | 0.008817 | 0.002939 | 2.70E-03 | AF | 0.7713 | | 0.012400 | 0.008200 | 1.30E-01 |
| rs4572913 | 4 | 46047009 | GABRA2 | CAAHTN | A | G | 0.8717 | -0.008435 | 0.003676 | 2.17E-02 | AF | 0.8501 | | 0.000500 | 0.010000 | 9.61E-01 |
| rs4389544 | 4 | 46741433 | GABRA2 | CAAHTN | C | T | 0.3306 | 0.006742 | 0.002636 | 1.06E-02 | AF | 0.3323 | | -0.000900 | 0.007100 | 9.01E-01 |
| rs10076365 | 5 | 161917408 | GABRA6 | CAAHTN | A | G | 0.8338 | -0.010038 | 0.003306 | 2.39E-03 | AF | 0.8126 | | -0.009800 | 0.008900 | 2.69E-01 |
| rs12416245 | 10 | 111938114 | ADRA2A | CAAHTN | G | T | 0.1873 | 0.008922 | 0.003152 | 4.64E-03 | AF | 0.1827 | | 0.020600 | 0.008600 | 1.66E-02 |
| rs76100968 | 10 | 112887951 | ADRA2A | CAAHTN | T | C | 0.0628 | 0.010218 | 0.005060 | 4.35E-02 | AF | 0.0656 | | -0.004600 | 0.015300 | 7.62E-01 |
| rs6585052 | 10 | 113046403 | ADRA2A | CAAHTN | T | C | 0.9879 | 0.026214 | 0.011265 | 2.00E-02 | AF | 0.9516 | | 0.034000 | 0.026900 | 2.06E-01 |
| rs4366419 | 10 | 113049557 | ADRA2A | CAAHTN | G | A | 0.9188 | 0.012867 | 0.004513 | 4.36E-03 | AF | 0.8939 | | 0.019600 | 0.011900 | 1.01E-01 |
| rs4918689 | 10 | 113518004 | ADRA2A | CAAHTN | C | A | 0.0706 | 0.012865 | 0.004822 | 7.63E-03 | AF | 0.0746 | | -0.022000 | 0.012800 | 8.47E-02 |
| rs80339013 | 15 | 26272751 | GABRA5 | CAAHTN | C | T | 0.1174 | 0.012881 | 0.003896 | 9.47E-04 | AF | 0.1181 | | -0.003000 | 0.010900 | 7.84E-01 |
| rs3751583 | 15 | 26806393 | GABRA5 | CAAHTN | C | T | 0.0711 | -0.010156 | 0.004812 | 3.48E-02 | AF | 0.0694 | | 0.000700 | 0.013500 | 9.60E-01 |

**Abbreviations:** AF, atrial fibrillation; CAAHTN, centrally acting antihypertensives; Chr, chromosome; EA, effect allele; EAF, effect allele frequency; OA, other allele; Pos, genomic position; Pval, p-value; SE, standard error; SNP, single nucleotide polymorphism.

**Table S12. Effect estimates for the associations of the genetic variants with calcium channel blockers and atrial fibrillation for the primary analyses**

|  | | | | **Exposure effect estimates** | | | | | | | | | **Outcome effect estimates** | | | |
| --- | --- | --- | --- | --- | --- | --- | --- | --- | --- | --- | --- | --- | --- | --- | --- | --- |
| **SNP** | **Chr** | **Pos** | **Gene** | **Trait** | **EA** | **OA** | **EAF** | **Beta** | **SE** | **Pval** | **Trait** | **EAF** | | **Beta** | **SE** | **Pval** |
| rs12145535 | 1 | 53693831 | CPT2 | CCBs | T | C | 0.3294 | 0.007678 | 0.002618 | 3.36E-03 | AF | 0.3345 | | -0.015400 | 0.007100 | 2.90E-02 |
| rs115904908 | 2 | 152300384 | CACNB4 | CCBs | T | C | 0.0138 | -0.039069 | 0.010547 | 2.12E-04 | AF | 0.0157 | | -0.004700 | 0.032200 | 8.84E-01 |
| rs2139419 | 2 | 152864208 | CACNB4 | CCBs | T | C | 0.6420 | 0.007193 | 0.002577 | 5.25E-03 | AF | 0.6195 | | 0.004400 | 0.006900 | 5.31E-01 |
| rs74471041 | 3 | 49542543 | CACNA2D2 | CCBs | G | T | 0.2588 | 0.006897 | 0.002806 | 1.40E-02 | AF | 0.2575 | | 0.001700 | 0.007600 | 8.24E-01 |
| rs34484573 | 3 | 49880399 | CACNA2D2 | CCBs | A | G | 0.1261 | -0.019720 | 0.003707 | 1.04E-07 | AF | 0.1216 | | -0.026800 | 0.010600 | 1.15E-02 |
| rs3806708 | 3 | 50306249 | CACNA2D2 | CCBs | C | T | 0.1178 | -0.008168 | 0.003821 | 3.26E-02 | AF | 0.1154 | | 0.007100 | 0.010500 | 4.97E-01 |
| rs146992327 | 3 | 53389332 | CACNA1D | CCBs | C | T | 0.0109 | 0.032990 | 0.012092 | 6.37E-03 | AF | 0.0102 | | -0.028700 | 0.041600 | 4.91E-01 |
| rs6445583 | 3 | 53562894 | CACNA1D | CCBs | A | G | 0.7458 | 0.013858 | 0.002823 | 9.19E-07 | AF | 0.7351 | | 0.016100 | 0.007800 | 3.79E-02 |
| rs79020595 | 3 | 53646994 | CACNA1D | CCBs | A | G | 0.0173 | -0.033992 | 0.010059 | 7.27E-04 | AF | 0.0195 | | -0.063700 | 0.030700 | 3.77E-02 |
| rs6797014 | 3 | 53808198 | CACNA1D | CCBs | T | C | 0.3717 | 0.007207 | 0.002551 | 4.73E-03 | AF | 0.3770 | | 0.011700 | 0.006900 | 8.90E-02 |
| rs4583644 | 3 | 54008751 | CACNA1D | CCBs | A | G | 0.0316 | -0.020720 | 0.007039 | 3.24E-03 | AF | 0.0309 | | 0.011700 | 0.018600 | 5.30E-01 |
| rs111969626 | 3 | 54505741 | CACNA1D | CCBs | A | G | 0.5318 | -0.005380 | 0.002468 | 2.93E-02 | AF | 0.5420 | | -0.000200 | 0.006800 | 9.75E-01 |
| rs76492099 | 7 | 81939205 | CACNA2D1 | CCBs | A | G | 0.0630 | -0.011911 | 0.005094 | 1.94E-02 | AF | 0.0570 | | -0.001100 | 0.014900 | 9.42E-01 |
| rs2488161 | 10 | 18299365 | CACNB2 | CCBs | G | A | 0.1611 | 0.008202 | 0.003537 | 2.04E-02 | AF | 0.1582 | | 0.005800 | 0.009600 | 5.44E-01 |
| rs117926523 | 10 | 18336903 | CACNB2 | CCBs | G | A | 0.0159 | 0.029206 | 0.010161 | 4.05E-03 | AF | 0.0169 | | 0.043500 | 0.028900 | 1.32E-01 |
| rs2488158 | 10 | 18370324 | CACNB2 | CCBs | A | G | 0.1660 | 0.006929 | 0.003314 | 3.66E-02 | AF | 0.1606 | | 0.003300 | 0.008900 | 7.07E-01 |
| rs140766681 | 10 | 18409497 | CACNB2 | CCBs | C | A | 0.0212 | 0.021714 | 0.008551 | 1.11E-02 | AF | 0.0209 | | 0.031500 | 0.024400 | 1.98E-01 |
| rs10764322 | 10 | 18437061 | CACNB2 | CCBs | G | A | 0.3107 | 0.013826 | 0.002665 | 2.12E-07 | AF | 0.3050 | | 0.013800 | 0.007200 | 5.55E-02 |
| rs1757234 | 10 | 18526451 | CACNB2 | CCBs | A | G | 0.1164 | -0.007722 | 0.003844 | 4.46E-02 | AF | 0.1158 | | -0.035900 | 0.010400 | 5.81E-04 |
| rs2255266 | 10 | 18529720 | CACNB2 | CCBs | C | T | 0.7938 | -0.007116 | 0.003047 | 1.95E-02 | AF | 0.7733 | | -0.013400 | 0.008100 | 9.74E-02 |
| rs184067968 | 10 | 18529765 | CACNB2 | CCBs | A | C | 0.0165 | -0.024487 | 0.010073 | 1.51E-02 | AF | 0.0158 | | -0.002000 | 0.031400 | 9.49E-01 |
| rs12416030 | 10 | 18789075 | CACNB2 | CCBs | C | T | 0.2017 | 0.010977 | 0.003106 | 4.10E-04 | AF | 0.2092 | | 0.013600 | 0.008200 | 9.80E-02 |
| rs10828906 | 10 | 18841795 | CACNB2 | CCBs | T | C | 0.7351 | 0.011224 | 0.002791 | 5.79E-05 | AF | 0.7247 | | 0.009700 | 0.007600 | 1.98E-01 |
| rs115101482 | 10 | 18853351 | CACNB2 | CCBs | A | G | 0.0138 | 0.030768 | 0.010604 | 3.71E-03 | AF | 0.0159 | | 0.034600 | 0.030300 | 2.54E-01 |
| rs7901566 | 10 | 18987813 | CACNB2 | CCBs | A | G | 0.7378 | 0.007510 | 0.002821 | 7.76E-03 | AF | 0.7152 | | -0.005300 | 0.007600 | 4.86E-01 |
| rs7112615 | 11 | 68530318 | CPT1A | CCBs | A | C | 0.9384 | 0.011939 | 0.005123 | 1.98E-02 | AF | 0.9154 | | -0.018900 | 0.013900 | 1.74E-01 |
| rs117842198 | 11 | 68709890 | CPT1A | CCBs | A | G | 0.0097 | -0.026501 | 0.012663 | 3.64E-02 | AF | 0.0117 | | 0.073900 | 0.035800 | 3.93E-02 |
| rs7129324 | 11 | 68894909 | CPT1A | CCBs | A | G | 0.0166 | -0.023583 | 0.010238 | 2.12E-02 | AF | 0.0140 | | -0.048700 | 0.034200 | 1.55E-01 |
| rs11605129 | 11 | 69050915 | CPT1A | CCBs | T | C | 0.0528 | -0.019100 | 0.005508 | 5.25E-04 | AF | 0.0666 | | -0.022700 | 0.013700 | 9.72E-02 |
| rs882193 | 12 | 2350620 | CACNA1C | CCBs | A | G | 0.6183 | 0.006066 | 0.002579 | 1.87E-02 | AF | 0.6014 | | 0.006600 | 0.006900 | 3.41E-01 |
| rs55909860 | 12 | 2507848 | CACNA1C | CCBs | T | C | 0.0340 | 0.018480 | 0.006971 | 8.03E-03 | AF | 0.0352 | | 0.013600 | 0.020900 | 5.17E-01 |
| rs4765935 | 12 | 2563347 | CACNA1C | CCBs | C | A | 0.6986 | 0.005752 | 0.002709 | 3.37E-02 | AF | 0.6882 | | -0.013200 | 0.007500 | 7.70E-02 |
| rs2453467 | 12 | 49239951 | CACNB3 | CCBs | T | C | 0.7883 | 0.006459 | 0.003034 | 3.33E-02 | AF | 0.7721 | | -0.002200 | 0.008500 | 7.94E-01 |
| rs12317778 | 12 | 50105637 | CACNB3 | CCBs | C | T | 0.0823 | -0.021217 | 0.004493 | 2.34E-06 | AF | 0.0794 | | -0.029000 | 0.012300 | 1.78E-02 |
| rs71380229 | 16 | 1034503 | CACNA1H | CCBs | C | A | 0.0791 | 0.010421 | 0.004584 | 2.30E-02 | AF | 0.0914 | | 0.004300 | 0.012500 | 7.31E-01 |
| rs12926678 | 16 | 1187931 | CACNA1H | CCBs | T | C | 0.3830 | -0.005138 | 0.002575 | 4.60E-02 | AF | 0.3715 | | -0.002300 | 0.007500 | 7.59E-01 |
| rs117177120 | 16 | 1238875 | CACNA1H | CCBs | G | T | 0.0573 | 0.018092 | 0.005430 | 8.63E-04 | AF | 0.0676 | | 0.007200 | 0.015700 | 6.48E-01 |
| rs4347630 | 16 | 1243557 | CACNA1H | CCBs | C | T | 0.8154 | 0.007868 | 0.003295 | 1.70E-02 | AF | 0.8104 | | -0.016100 | 0.010300 | 1.16E-01 |
| rs8053994 | 16 | 1244201 | CACNA1H | CCBs | A | G | 0.5550 | -0.006127 | 0.002537 | 1.57E-02 | AF | 0.5449 | | -0.013400 | 0.007400 | 6.86E-02 |
| rs1977100 | 16 | 1310921 | CACNA1H | CCBs | G | A | 0.3203 | -0.006019 | 0.002638 | 2.25E-02 | AF | 0.3283 | | -0.011800 | 0.007400 | 1.14E-01 |
| rs2338115 | 17 | 36929578 | CACNB1 | CCBs | T | C | 0.5544 | -0.005892 | 0.002476 | 1.73E-02 | AF | 0.5363 | | 0.004500 | 0.006200 | 4.64E-01 |
| rs71369724 | 17 | 37326350 | CACNB1 | CCBs | A | G | 0.0540 | 0.011835 | 0.005465 | 3.03E-02 | AF | 0.0507 | | -0.016000 | 0.017400 | 3.59E-01 |
| rs61554907 | 17 | 38220432 | CACNB1 | CCBs | T | G | 0.1095 | 0.008199 | 0.003974 | 3.91E-02 | AF | 0.1185 | | 0.025900 | 0.010600 | 1.47E-02 |
| rs8065903 | 17 | 48629458 | CACNA1G | CCBs | G | A | 0.7381 | -0.007571 | 0.002795 | 6.76E-03 | AF | 0.7218 | | -0.015300 | 0.007600 | 4.29E-02 |
| rs198535 | 17 | 48635052 | CACNA1G | CCBs | G | A | 0.4177 | -0.011022 | 0.002506 | 1.10E-05 | AF | 0.4239 | | -0.005900 | 0.006800 | 3.85E-01 |
| rs34157595 | 17 | 49200947 | CACNA1G | CCBs | C | T | 0.3950 | -0.005087 | 0.002532 | 4.45E-02 | AF | 0.4041 | | -0.001400 | 0.007000 | 8.40E-01 |
| rs78784512 | 17 | 64195835 | CACNG1 | CCBs | T | C | 0.0228 | 0.016976 | 0.008618 | 4.89E-02 | AF | 0.0215 | | -0.012200 | 0.026900 | 6.50E-01 |
| rs136832 | 22 | 40046538 | CACNA1I | CCBs | T | C | 0.1864 | 0.006252 | 0.003158 | 4.77E-02 | AF | 0.1896 | | 0.011100 | 0.008600 | 1.94E-01 |
| rs117741951 | 22 | 40403113 | CACNA1I | CCBs | T | C | 0.0294 | -0.018231 | 0.007466 | 1.46E-02 | AF | 0.0291 | | 0.017500 | 0.023800 | 4.62E-01 |

**Abbreviations:** AF, atrial fibrillation; CCBs, calcium channel blockers; Chr, chromosome; EA, effect allele; EAF, effect allele frequency; OA, other allele; Pos, genomic position; Pval, p-value; SE, standard error; SNP, single nucleotide polymorphism.

**Table S13. Effect estimates for the associations of the genetic variants with loop diuretics and atrial fibrillation for the primary analyses**

|  | | | | **Exposure effect estimates** | | | | | | | | | **Outcome effect estimates** | | | |
| --- | --- | --- | --- | --- | --- | --- | --- | --- | --- | --- | --- | --- | --- | --- | --- | --- |
| **SNP** | **Chr** | **Pos** | **Gene** | **Trait** | **EA** | **OA** | **EAF** | **Beta** | **SE** | **Pval** | **Trait** | **EAF** | | **Beta** | **SE** | **Pval** |
| rs10067219 | 5 | 127095999 | SLC12A2 | LDs | C | T | 0.7458 | -0.007666 | 0.002829 | 6.73E-03 | AF | 0.7252 | | 0.001400 | 0.007700 | 8.52E-01 |
| rs2409040 | 5 | 127291878 | SLC12A2 | LDs | C | T | 0.5496 | 0.006897 | 0.002474 | 5.31E-03 | AF | 0.5532 | | 0.005300 | 0.006700 | 4.31E-01 |
| rs78736765 | 5 | 127693997 | SLC12A2 | LDs | T | C | 0.1345 | -0.011330 | 0.003622 | 1.76E-03 | AF | 0.1458 | | -0.021200 | 0.009500 | 2.54E-02 |
| rs12914000 | 15 | 47906718 | SLC12A1 | LDs | C | T | 0.1631 | -0.006925 | 0.003383 | 4.07E-02 | AF | 0.1569 | | 0.000100 | 0.009400 | 9.89E-01 |
| rs2903908 | 20 | 44693947 | SLC12A5 | LDs | C | T | 0.2605 | 0.007690 | 0.002811 | 6.23E-03 | AF | 0.2648 | | -0.012000 | 0.007600 | 1.14E-01 |

**Abbreviations:** AF, atrial fibrillation; Chr, chromosome; EA, effect allele; EAF, effect allele frequency; LDs, loop diuretics; OA, other allele; Pos, genomic position; Pval, p-value; SE, standard error; SNP, single nucleotide polymorphism.

**Table S14. Effect estimates for the associations of the genetic variants with potassium-sparing diuretics and mineralocorticoid receptor antagonists and atrial fibrillation for the primary analyses**

|  | | | | **Exposure effect estimates** | | | | | | | | | **Outcome effect estimates** | | | |
| --- | --- | --- | --- | --- | --- | --- | --- | --- | --- | --- | --- | --- | --- | --- | --- | --- |
| **SNP** | **Chr** | **Pos** | **Gene** | **Trait** | **EA** | **OA** | **EAF** | **Beta** | **SE** | **Pval** | **Trait** | **EAF** | | **Beta** | **SE** | **Pval** |
| rs12079515 | 1 | 1215712 | SCNN1D | PSDs and MRAs | T | C | 0.0376 | -0.021206 | 0.006502 | 1.11E-03 | AF | 0.0367 | | -0.005900 | 0.018400 | 7.47E-01 |
| rs12746510 | 1 | 1319123 | SCNN1D | PSDs and MRAs | C | T | 0.1195 | -0.008132 | 0.003800 | 3.24E-02 | AF | 0.1175 | | -0.011900 | 0.011000 | 2.80E-01 |
| rs13303195 | 1 | 1922303 | SCNN1D | PSDs and MRAs | T | C | 0.5316 | 0.007156 | 0.002463 | 3.67E-03 | AF | 0.5356 | | 0.004700 | 0.007700 | 5.40E-01 |
| rs71616586 | 4 | 149398316 | NR3C2 | PSDs and MRAs | A | G | 0.0661 | 0.011804 | 0.004949 | 1.71E-02 | AF | 0.0665 | | 0.009500 | 0.015100 | 5.27E-01 |
| rs150274214 | 16 | 22727592 | SCNN1G | PSDs and MRAs | A | G | 0.0193 | 0.023979 | 0.009202 | 9.16E-03 | AF | 0.0174 | | -0.018800 | 0.032100 | 5.59E-01 |
| rs9931863 | 16 | 23249779 | SCNN1B | PSDs and MRAs | G | A | 0.2436 | 0.006084 | 0.002892 | 3.54E-02 | AF | 0.2572 | | -0.008900 | 0.007900 | 2.61E-01 |
| rs2520014 | 16 | 23794098 | SCNN1B | PSDs and MRAs | A | G | 0.4266 | -0.005332 | 0.002487 | 3.20E-02 | AF | 0.4266 | | 0.003500 | 0.006700 | 6.03E-01 |

**Abbreviations:** AF, atrial fibrillation; Chr, chromosome; EA, effect allele; EAF, effect allele frequency; MRAs, mineralocorticoid receptor antagonists; OA, other allele; Pos, genomic position; PSDs, potassium sparing diuretics; Pval, p-value; SE, standard error; SNP, single nucleotide polymorphism.

**Table S15. Effect estimates for the associations of the genetic variants with renin inhibitors and atrial fibrillation for the primary analyses**

|  | | | | **Exposure effect estimates** | | | | | | | | | **Outcome effect estimates** | | | |
| --- | --- | --- | --- | --- | --- | --- | --- | --- | --- | --- | --- | --- | --- | --- | --- | --- |
| **SNP** | **Chr** | **Pos** | **Gene** | **Trait** | **EA** | **OA** | **EAF** | **Beta** | **SE** | **Pval** | **Trait** | **EAF** | | **Beta** | **SE** | **Pval** |
| rs6668579 | 1 | 203330876 | REN | RIs | A | C | 0.9822 | 0.019187 | 0.009534 | 4.42E-02 | AF | 0.9609 | | -0.016500 | 0.027700 | 5.51E-01 |
| rs74650517 | 1 | 204467415 | REN | RIs | A | G | 0.0179 | 0.018466 | 0.009300 | 4.71E-02 | AF | 0.0165 | | 0.027600 | 0.028100 | 3.25E-01 |

**Abbreviations:** AF, atrial fibrillation; Chr, chromosome; EA, effect allele; EAF, effect allele frequency; OA, other allele; Pos, genomic position; Pval, p-value; RIs; renin inhibitors; SE, standard error; SNP, single nucleotide polymorphism.

**Table S16. Effect estimates for the associations of the genetic variants with thiazides and related diuretic agents and atrial fibrillation for the primary analyses**

|  | | | | **Exposure effect estimates** | | | | | | | | | **Outcome effect estimates** | | | |
| --- | --- | --- | --- | --- | --- | --- | --- | --- | --- | --- | --- | --- | --- | --- | --- | --- |
| **SNP** | **Chr** | **Pos** | **Gene** | **Trait** | **EA** | **OA** | **EAF** | **Beta** | **SE** | **Pval** | **Trait** | **EAF** | | **Beta** | **SE** | **Pval** |
| rs191430682 | 1 | 1348003 | GABRD | Thiazides | G | A | 0.0357 | -0.030360 | 0.006665 | 5.24E-06 | AF | 0.0359 | | 0.002800 | 0.020000 | 8.88E-01 |
| rs2076327 | 1 | 1686962 | GABRD | Thiazides | T | C | 0.4926 | 0.011040 | 0.002481 | 8.63E-06 | AF | 0.5037 | | 0.014100 | 0.007200 | 5.04E-02 |
| rs116733754 | 1 | 1721556 | GABRD | Thiazides | A | G | 0.0124 | -0.036678 | 0.011107 | 9.59E-04 | AF | 0.0112 | | -0.013300 | 0.040500 | 7.42E-01 |
| rs72911372 | 1 | 1950551 | GABRD | Thiazides | A | G | 0.1696 | 0.007795 | 0.003484 | 2.53E-02 | AF | 0.1788 | | 0.014700 | 0.010100 | 1.47E-01 |
| rs4648614 | 1 | 1953812 | GABRD | Thiazides | T | C | 0.6891 | 0.006111 | 0.002693 | 2.33E-02 | AF | 0.6799 | | 0.001300 | 0.007900 | 8.65E-01 |
| rs12746525 | 1 | 2895806 | GABRD | Thiazides | T | C | 0.6384 | -0.005496 | 0.002586 | 3.36E-02 | AF | 0.6190 | | 0.008000 | 0.007200 | 2.70E-01 |
| rs139787011 | 4 | 45844166 | GABRG1 | Thiazides | G | A | 0.0165 | 0.021613 | 0.009930 | 2.95E-02 | AF | 0.0231 | | -0.006100 | 0.028300 | 8.28E-01 |
| rs7699135 | 4 | 45956676 | GABRG1 | Thiazides | T | C | 0.8676 | -0.009593 | 0.003649 | 8.57E-03 | AF | 0.8462 | | -0.006000 | 0.009900 | 5.40E-01 |
| rs79102205 | 4 | 46475738 | GABRB1 | Thiazides | C | T | 0.0480 | 0.012485 | 0.005808 | 3.16E-02 | AF | 0.0448 | | -0.022600 | 0.017700 | 2.02E-01 |
| rs4389544 | 4 | 46741433 | GABRA2 | Thiazides | C | T | 0.3306 | 0.006742 | 0.002636 | 1.06E-02 | AF | 0.3323 | | -0.000900 | 0.007100 | 9.01E-01 |
| rs17446772 | 5 | 160551096 | GABRG2 | Thiazides | A | C | 0.0154 | -0.021140 | 0.010060 | 3.56E-02 | AF | 0.0148 | | 0.032800 | 0.032300 | 3.10E-01 |
| rs10076365 | 5 | 161917408 | GABRA6 | Thiazides | A | G | 0.8338 | -0.010038 | 0.003306 | 2.39E-03 | AF | 0.8126 | | -0.009800 | 0.008900 | 2.69E-01 |
| rs142415851 | 5 | 170016193 | GABRP | Thiazides | T | C | 0.0302 | 0.016568 | 0.007513 | 2.74E-02 | AF | 0.0293 | | -0.007400 | 0.023400 | 7.52E-01 |
| rs79547497 | 5 | 171063750 | GABRP | Thiazides | C | T | 0.0298 | 0.019308 | 0.007479 | 9.83E-03 | AF | 0.0260 | | 0.014100 | 0.025300 | 5.77E-01 |
| rs73318453 | 5 | 171095180 | GABRP | Thiazides | G | T | 0.0215 | 0.022324 | 0.008440 | 8.17E-03 | AF | 0.0242 | | -0.010100 | 0.021500 | 6.41E-01 |
| rs111617018 | 8 | 86420751 | CA1 | Thiazides | A | G | 0.0687 | -0.011737 | 0.004873 | 1.60E-02 | AF | 0.0671 | | 0.001000 | 0.015700 | 9.47E-01 |
| rs62509890 | 8 | 87064009 | CA1 | Thiazides | G | A | 0.1141 | 0.010297 | 0.003902 | 8.31E-03 | AF | 0.1137 | | -0.003700 | 0.010800 | 7.30E-01 |
| rs72686797 | 8 | 87215470 | CA2 | Thiazides | C | A | 0.1017 | -0.010933 | 0.004254 | 1.02E-02 | AF | 0.1107 | | 0.012500 | 0.012000 | 3.00E-01 |
| rs80339013 | 15 | 26272751 | GABRA5 | Thiazides | C | T | 0.1174 | 0.012881 | 0.003896 | 9.47E-04 | AF | 0.1181 | | -0.003000 | 0.010900 | 7.84E-01 |
| rs8030011 | 15 | 26818362 | GABRB3 | Thiazides | A | G | 0.1292 | -0.008237 | 0.003677 | 2.51E-02 | AF | 0.1268 | | 0.011800 | 0.009900 | 2.34E-01 |
| rs117280013 | 15 | 26969128 | GABRG3 | Thiazides | A | G | 0.0431 | -0.012372 | 0.006068 | 4.15E-02 | AF | 0.0539 | | 0.001800 | 0.016800 | 9.16E-01 |
| rs140443467 | 15 | 27722954 | GABRG3 | Thiazides | G | A | 0.0281 | -0.029713 | 0.007696 | 1.13E-04 | AF | 0.0318 | | 0.007800 | 0.020500 | 7.02E-01 |
| rs12914000 | 15 | 47906718 | SLC12A1 | Thiazides | C | T | 0.1631 | -0.006925 | 0.003383 | 4.07E-02 | AF | 0.1569 | | 0.000100 | 0.009400 | 9.89E-01 |

**Abbreviations:** AF, atrial fibrillation; Chr, chromosome; EA, effect allele; EAF, effect allele frequency; OA, other allele; Pos, genomic position; Pval, p-value; SE, standard error; SNP, single nucleotide polymorphism; Thiazides; thiazides and related diuretics.

**Table S17. Effect estimates for the associations of the genetic variants with vasodilators and atrial fibrillation for the primary analyses**

|  | | | | **Exposure effect estimates** | | | | | | | | | **Outcome effect estimates** | | | |
| --- | --- | --- | --- | --- | --- | --- | --- | --- | --- | --- | --- | --- | --- | --- | --- | --- |
| **SNP** | **Chr** | **Pos** | **Gene** | **Trait** | **EA** | **OA** | **EAF** | **Beta** | **SE** | **Pval** | **Trait** | **EAF** | | **Beta** | **SE** | **Pval** |
| rs3795393 | 1 | 153509890 | NPR1 | VDs | C | T | 0.0662 | -0.009861 | 0.004946 | 4.62E-02 | AF | 0.0652 | | -0.010900 | 0.014000 | 4.37E-01 |
| rs4845568 | 1 | 153704034 | NPR1 | VDs | C | T | 0.9475 | 0.015022 | 0.005515 | 6.45E-03 | AF | 0.9186 | | 0.026700 | 0.015200 | 7.88E-02 |
| rs4845626 | 1 | 154423485 | NPR1 | VDs | T | G | 0.1676 | -0.007240 | 0.003295 | 2.80E-02 | AF | 0.1881 | | -0.004200 | 0.008500 | 6.19E-01 |
| rs73842253 | 4 | 119721856 | PDE5A | VDs | A | G | 0.0636 | -0.011261 | 0.005053 | 2.59E-02 | AF | 0.0655 | | -0.005200 | 0.013600 | 7.03E-01 |
| rs2714978 | 4 | 120670213 | PDE5A | VDs | G | A | 0.4020 | 0.006845 | 0.002510 | 6.40E-03 | AF | 0.4039 | | 0.001500 | 0.006700 | 8.23E-01 |
| rs10305838 | 4 | 148400256 | EDNRA | VDs | C | T | 0.1399 | 0.012965 | 0.003543 | 2.53E-04 | AF | 0.1380 | | 0.017400 | 0.009500 | 6.64E-02 |
| rs62509890 | 8 | 87064009 | CA1 | VDs | G | A | 0.1141 | 0.010297 | 0.003902 | 8.31E-03 | AF | 0.1137 | | -0.003700 | 0.010800 | 7.30E-01 |
| rs11023983 | 11 | 16566923 | KCNJ11 | VDs | C | A | 0.4700 | -0.007226 | 0.002462 | 3.33E-03 | AF | 0.4641 | | -0.008600 | 0.006700 | 1.97E-01 |
| rs2074311 | 11 | 17421860 | KCNJ11 | VDs | G | A | 0.5858 | -0.009240 | 0.002495 | 2.13E-04 | AF | 0.5642 | | -0.002100 | 0.006700 | 7.57E-01 |
| rs10766408 | 11 | 17539212 | KCNJ11 | VDs | C | T | 0.6240 | -0.005390 | 0.002543 | 3.40E-02 | AF | 0.6094 | | -0.000400 | 0.006800 | 9.57E-01 |
| rs12287965 | 11 | 17553706 | KCNJ11 | VDs | T | C | 0.0813 | 0.010434 | 0.004489 | 2.01E-02 | AF | 0.0790 | | 0.007500 | 0.012400 | 5.48E-01 |
| rs73569213 | 11 | 128478380 | KCNJ1 | VDs | A | G | 0.1779 | -0.006528 | 0.003213 | 4.22E-02 | AF | 0.1792 | | -0.000400 | 0.008800 | 9.64E-01 |
| rs731152 | 17 | 40279680 | AOC3 | VDs | A | G | 0.2268 | -0.006931 | 0.002934 | 1.82E-02 | AF | 0.2322 | | 0.015000 | 0.007800 | 5.50E-02 |
| rs138643143 | 17 | 40709867 | AOC3 | VDs | A | G | 0.0750 | 0.019321 | 0.004962 | 9.89E-05 | AF | 0.0747 | | 0.027800 | 0.015100 | 6.68E-02 |
| rs74754758 | 17 | 40902035 | AOC3 | VDs | C | T | 0.0170 | -0.035157 | 0.009820 | 3.44E-04 | AF | 0.0183 | | -0.025600 | 0.028000 | 3.62E-01 |
| rs2241359 | 19 | 14586245 | PTGER1 | VDs | A | G | 0.1615 | 0.007838 | 0.003344 | 1.91E-02 | AF | 0.1608 | | 0.005200 | 0.009300 | 5.80E-01 |
| rs3760702 | 19 | 14588237 | PTGER1 | VDs | A | G | 0.3302 | -0.005445 | 0.002617 | 3.75E-02 | AF | 0.3221 | | -0.007900 | 0.007400 | 2.90E-01 |
| rs62111755 | 19 | 46253766 | PTGIR | VDs | G | T | 0.1523 | 0.008838 | 0.003415 | 9.66E-03 | AF | 0.1578 | | 0.005500 | 0.009100 | 5.44E-01 |
| rs138809535 | 19 | 46279757 | PTGIR | VDs | T | C | 0.0192 | 0.017792 | 0.008998 | 4.80E-02 | AF | 0.0186 | | 0.002300 | 0.027400 | 9.34E-01 |

**Abbreviations:** AF, atrial fibrillation; Chr, chromosome; EA, effect allele; EAF, effect allele frequency; OA, other allele; Pos, genomic position; Pval, p-value; SE, standard error; SNP, single nucleotide polymorphism; VDs, vasodilators.

**Table S18. Effect estimates for the associations of the genetic variants with all 12 antihypertensive drug classes combined and atrial fibrillation for the primary analyses**

|  | | | | **Exposure effect estimates** | | | | | | | | | **Outcome effect estimates** | | | |
| --- | --- | --- | --- | --- | --- | --- | --- | --- | --- | --- | --- | --- | --- | --- | --- | --- |
| **SNP** | **Chr** | **Pos** | **Gene** | **Trait** | **EA** | **OA** | **EAF** | **Beta** | **SE** | **Pval** | **Trait** | **EAF** | | **Beta** | **SE** | **Pval** |
| rs12079515 | 1 | 1215712 | SCNN1D | AntiHTN | T | C | 0.0376 | -0.021206 | 0.006502 | 1.11E-03 | AF | 0.0367 | | -0.005900 | 0.018400 | 7.47E-01 |
| rs12746510 | 1 | 1319123 | SCNN1D | AntiHTN | C | T | 0.1195 | -0.008132 | 0.003800 | 3.24E-02 | AF | 0.1175 | | -0.011900 | 0.011000 | 2.80E-01 |
| rs191430682 | 1 | 1348003 | GABRD | AntiHTN | G | A | 0.0357 | -0.030360 | 0.006665 | 5.24E-06 | AF | 0.0359 | | 0.002800 | 0.020000 | 8.88E-01 |
| rs2076327 | 1 | 1686962 | GABRD | AntiHTN | T | C | 0.4926 | 0.011040 | 0.002481 | 8.63E-06 | AF | 0.5037 | | 0.014100 | 0.007200 | 5.04E-02 |
| rs116733754 | 1 | 1721556 | GABRD | AntiHTN | A | G | 0.0124 | -0.036678 | 0.011107 | 9.59E-04 | AF | 0.0112 | | -0.013300 | 0.040500 | 7.42E-01 |
| rs13303195 | 1 | 1922303 | SCNN1D | AntiHTN | T | C | 0.5316 | 0.007156 | 0.002463 | 3.67E-03 | AF | 0.5356 | | 0.004700 | 0.007700 | 5.40E-01 |
| rs72911372 | 1 | 1950551 | GABRD | AntiHTN | A | G | 0.1696 | 0.007795 | 0.003484 | 2.53E-02 | AF | 0.1788 | | 0.014700 | 0.010100 | 1.47E-01 |
| rs4648614 | 1 | 1953812 | GABRD | AntiHTN | T | C | 0.6891 | 0.006111 | 0.002693 | 2.33E-02 | AF | 0.6799 | | 0.001300 | 0.007900 | 8.65E-01 |
| rs12746525 | 1 | 2895806 | GABRD | AntiHTN | T | C | 0.6384 | -0.005496 | 0.002586 | 3.36E-02 | AF | 0.6190 | | 0.008000 | 0.007200 | 2.70E-01 |
| rs12145535 | 1 | 53693831 | CPT2 | AntiHTN | T | C | 0.3294 | 0.007678 | 0.002618 | 3.36E-03 | AF | 0.3345 | | -0.015400 | 0.007100 | 2.90E-02 |
| rs3795393 | 1 | 153509890 | NPR1 | AntiHTN | C | T | 0.0662 | -0.009861 | 0.004946 | 4.62E-02 | AF | 0.0652 | | -0.010900 | 0.014000 | 4.37E-01 |
| rs4845568 | 1 | 153704034 | NPR1 | AntiHTN | C | T | 0.9475 | 0.015022 | 0.005515 | 6.45E-03 | AF | 0.9186 | | 0.026700 | 0.013800 | 3.92E-01 |
| rs4845568 | 1 | 153704034 | NPR1 | AntiHTN | C | T | 0.9475 | 0.015022 | 0.005515 | 6.45E-03 | AF | 0.9186 | | 0.026700 | 0.015200 | 7.88E-02 |
| rs60715601 | 1 | 154002788 | NPR1 | AntiHTN | C | A | 0.0614 | 0.011826 | 0.005135 | 2.13E-02 | AF | 0.0577 | | -0.003000 | 0.014400 | 8.36E-01 |
| rs4845626 | 1 | 154423485 | NPR1 | AntiHTN | T | G | 0.1676 | -0.007240 | 0.003295 | 2.80E-02 | AF | 0.1881 | | -0.004200 | 0.008500 | 6.19E-01 |
| rs6668579 | 1 | 203330876 | REN | AntiHTN | A | C | 0.9822 | 0.019187 | 0.009534 | 4.42E-02 | AF | 0.9609 | | -0.016500 | 0.027700 | 5.51E-01 |
| rs74650517 | 1 | 204467415 | REN | AntiHTN | A | G | 0.0179 | 0.018466 | 0.009300 | 4.71E-02 | AF | 0.0165 | | 0.027600 | 0.028100 | 3.25E-01 |
| rs62153010 | 2 | 96854568 | ADRA2B | AntiHTN | A | G | 0.1699 | -0.010002 | 0.003277 | 2.27E-03 | AF | 0.1697 | | 0.009900 | 0.008900 | 2.66E-01 |
| rs115904908 | 2 | 152300384 | CACNB4 | AntiHTN | T | C | 0.0138 | -0.039069 | 0.010547 | 2.12E-04 | AF | 0.0157 | | -0.004700 | 0.032200 | 8.84E-01 |
| rs2139419 | 2 | 152864208 | CACNB4 | AntiHTN | T | C | 0.6420 | 0.007193 | 0.002577 | 5.25E-03 | AF | 0.6195 | | 0.004400 | 0.006900 | 5.31E-01 |
| rs9829399 | 3 | 11652673 | PPARG | AntiHTN | T | C | 0.1172 | 0.008104 | 0.003822 | 3.40E-02 | AF | 0.1211 | | 0.000400 | 0.010000 | 9.65E-01 |
| rs74471041 | 3 | 49542543 | CACNA2D2 | AntiHTN | G | T | 0.2588 | 0.006897 | 0.002806 | 1.40E-02 | AF | 0.2575 | | 0.001700 | 0.007600 | 8.24E-01 |
| rs34484573 | 3 | 49880399 | CACNA2D2 | AntiHTN | A | G | 0.1261 | -0.019720 | 0.003707 | 1.04E-07 | AF | 0.1216 | | -0.026800 | 0.010600 | 1.15E-02 |
| rs3806708 | 3 | 50306249 | CACNA2D2 | AntiHTN | C | T | 0.1178 | -0.008168 | 0.003821 | 3.26E-02 | AF | 0.1154 | | 0.007100 | 0.010500 | 4.97E-01 |
| rs524986 | 3 | 52228496 | NISCH | AntiHTN | C | T | 0.5333 | 0.007881 | 0.002466 | 1.39E-03 | AF | 0.5444 | | -0.000200 | 0.006700 | 9.74E-01 |
| rs149028979 | 3 | 53132630 | NISCH | AntiHTN | A | G | 0.0460 | 0.023519 | 0.005998 | 8.82E-05 | AF | 0.0436 | | 0.035300 | 0.017700 | 4.63E-02 |
| rs7610701 | 3 | 53176264 | NISCH | AntiHTN | G | T | 0.5785 | 0.007200 | 0.002693 | 7.51E-03 | AF | 0.5836 | | -0.006600 | 0.007600 | 3.84E-01 |
| rs6764111 | 3 | 53207401 | NISCH | AntiHTN | G | A | 0.7725 | 0.008817 | 0.002939 | 2.70E-03 | AF | 0.7713 | | 0.012400 | 0.008200 | 1.30E-01 |
| rs146992327 | 3 | 53389332 | CACNA1D | AntiHTN | C | T | 0.0109 | 0.032990 | 0.012092 | 6.37E-03 | AF | 0.0102 | | -0.028700 | 0.041600 | 4.91E-01 |
| rs6445583 | 3 | 53562894 | CACNA1D | AntiHTN | A | G | 0.7458 | 0.013858 | 0.002823 | 9.19E-07 | AF | 0.7351 | | 0.016100 | 0.007800 | 3.79E-02 |
| rs79020595 | 3 | 53646994 | CACNA1D | AntiHTN | A | G | 0.0173 | -0.033992 | 0.010059 | 7.27E-04 | AF | 0.0195 | | -0.063700 | 0.030700 | 3.77E-02 |
| rs6797014 | 3 | 53808198 | CACNA1D | AntiHTN | T | C | 0.3717 | 0.007207 | 0.002551 | 4.73E-03 | AF | 0.3770 | | 0.011700 | 0.006900 | 8.90E-02 |
| rs4583644 | 3 | 54008751 | CACNA1D | AntiHTN | A | G | 0.0316 | -0.020720 | 0.007039 | 3.24E-03 | AF | 0.0309 | | 0.011700 | 0.018600 | 5.30E-01 |
| rs111969626 | 3 | 54505741 | CACNA1D | AntiHTN | A | G | 0.5318 | -0.005380 | 0.002468 | 2.93E-02 | AF | 0.5420 | | -0.000200 | 0.006800 | 9.75E-01 |
| rs12489143 | 3 | 148105556 | AGTR1 | AntiHTN | G | A | 0.0425 | 0.012518 | 0.006082 | 3.96E-02 | AF | 0.0434 | | -0.006200 | 0.015400 | 6.89E-01 |
| rs59252002 | 3 | 148137212 | AGTR1 | AntiHTN | C | T | 0.0976 | 0.008680 | 0.004141 | 3.61E-02 | AF | 0.0984 | | -0.020600 | 0.011100 | 6.39E-02 |
| rs80350379 | 3 | 148485148 | AGTR1 | AntiHTN | T | C | 0.0167 | -0.021258 | 0.009573 | 2.64E-02 | AF | 0.0293 | | 0.041100 | 0.025300 | 1.05E-01 |
| rs73015088 | 3 | 148832218 | AGTR1 | AntiHTN | T | C | 0.0076 | -0.030585 | 0.014234 | 3.17E-02 | AF | 0.0068 | | -0.030600 | 0.038300 | 4.25E-01 |
| rs118123032 | 3 | 148913426 | AGTR1 | AntiHTN | T | C | 0.0328 | -0.022378 | 0.006917 | 1.22E-03 | AF | 0.0355 | | -0.040600 | 0.019100 | 3.35E-02 |
| rs79387447 | 3 | 149370293 | AGTR1 | AntiHTN | A | G | 0.0134 | -0.021610 | 0.010723 | 4.39E-02 | AF | 0.0130 | | 0.039700 | 0.034400 | 2.49E-01 |
| rs139787011 | 4 | 45844166 | GABRG1 | AntiHTN | G | A | 0.0165 | 0.021613 | 0.009930 | 2.95E-02 | AF | 0.0231 | | -0.006100 | 0.028300 | 8.28E-01 |
| rs7699135 | 4 | 45956676 | GABRG1 | AntiHTN | T | C | 0.8676 | -0.009593 | 0.003649 | 8.57E-03 | AF | 0.8462 | | -0.006000 | 0.009900 | 5.40E-01 |
| rs79102205 | 4 | 46475738 | GABRB1 | AntiHTN | C | T | 0.0480 | 0.012485 | 0.005808 | 3.16E-02 | AF | 0.0448 | | -0.022600 | 0.017700 | 2.02E-01 |
| rs4389544 | 4 | 46741433 | GABRA2 | AntiHTN | C | T | 0.3306 | 0.006742 | 0.002636 | 1.06E-02 | AF | 0.3323 | | -0.000900 | 0.007100 | 9.01E-01 |
| rs73842253 | 4 | 119721856 | PDE5A | AntiHTN | A | G | 0.0636 | -0.011261 | 0.005053 | 2.59E-02 | AF | 0.0655 | | -0.005200 | 0.013600 | 7.03E-01 |
| rs2714978 | 4 | 120670213 | PDE5A | AntiHTN | G | A | 0.4020 | 0.006845 | 0.002510 | 6.40E-03 | AF | 0.4039 | | 0.001500 | 0.006700 | 8.23E-01 |
| rs10305838 | 4 | 148400256 | EDNRA | AntiHTN | C | T | 0.1399 | 0.012965 | 0.003543 | 2.53E-04 | AF | 0.1380 | | 0.017400 | 0.009500 | 6.64E-02 |
| rs71616586 | 4 | 149398316 | NR3C2 | AntiHTN | A | G | 0.0661 | 0.011804 | 0.004949 | 1.71E-02 | AF | 0.0665 | | 0.009500 | 0.015100 | 5.27E-01 |
| rs10067219 | 5 | 127095999 | SLC12A2 | AntiHTN | C | T | 0.7458 | -0.007666 | 0.002829 | 6.73E-03 | AF | 0.7252 | | 0.001400 | 0.007700 | 8.52E-01 |
| rs2409040 | 5 | 127291878 | SLC12A2 | AntiHTN | C | T | 0.5496 | 0.006897 | 0.002474 | 5.31E-03 | AF | 0.5532 | | 0.005300 | 0.006700 | 4.31E-01 |
| rs78736765 | 5 | 127693997 | SLC12A2 | AntiHTN | T | C | 0.1345 | -0.011330 | 0.003622 | 1.76E-03 | AF | 0.1458 | | -0.021200 | 0.009500 | 2.54E-02 |
| rs115412659 | 5 | 148150080 | ADRB2 | AntiHTN | A | G | 0.0582 | 0.012452 | 0.005290 | 1.86E-02 | AF | 0.0507 | | 0.034200 | 0.017000 | 4.35E-02 |
| rs7737361 | 5 | 148346443 | ADRB2 | AntiHTN | A | G | 0.1913 | -0.014132 | 0.003178 | 8.74E-06 | AF | 0.2032 | | 0.005900 | 0.008300 | 4.73E-01 |
| rs10067003 | 5 | 148547490 | ADRB2 | AntiHTN | T | C | 0.2422 | 0.008417 | 0.002905 | 3.77E-03 | AF | 0.2415 | | -0.005100 | 0.007900 | 5.22E-01 |
| rs4705073 | 5 | 148832326 | ADRB2 | AntiHTN | C | T | 0.4976 | 0.006751 | 0.002473 | 6.34E-03 | AF | 0.5043 | | -0.001700 | 0.006700 | 7.98E-01 |
| rs11750184 | 5 | 148846518 | ADRB2 | AntiHTN | A | G | 0.2665 | -0.006937 | 0.002791 | 1.30E-02 | AF | 0.2615 | | -0.005300 | 0.007500 | 4.80E-01 |
| rs17645325 | 5 | 158476709 | ADRA1B | AntiHTN | C | T | 0.1251 | 0.014443 | 0.003713 | 1.00E-04 | AF | 0.1268 | | 0.006600 | 0.010200 | 5.18E-01 |
| rs13159674 | 5 | 158643137 | ADRA1B | AntiHTN | T | C | 0.2021 | 0.008472 | 0.003063 | 5.67E-03 | AF | 0.2056 | | -0.002400 | 0.008300 | 7.68E-01 |
| rs12654978 | 5 | 159006314 | ADRA1B | AntiHTN | T | C | 0.3464 | -0.005126 | 0.002594 | 4.82E-02 | AF | 0.3260 | | -0.000600 | 0.007200 | 9.35E-01 |
| rs1009890 | 5 | 159329699 | ADRA1B | AntiHTN | C | T | 0.6855 | 0.005230 | 0.002657 | 4.90E-02 | AF | 0.6670 | | -0.003500 | 0.007200 | 6.30E-01 |
| rs143589638 | 5 | 159629575 | ADRA1B | AntiHTN | G | A | 0.0520 | -0.011825 | 0.005550 | 3.31E-02 | AF | 0.0486 | | -0.011200 | 0.015300 | 4.66E-01 |
| rs13188637 | 5 | 160335398 | GABRB2 | AntiHTN | A | G | 0.5032 | -0.004941 | 0.002465 | 4.50E-02 | AF | 0.4956 | | -0.021300 | 0.006700 | 1.40E-03 |
| rs17446772 | 5 | 160551096 | GABRG2 | AntiHTN | A | C | 0.0154 | -0.021140 | 0.010060 | 3.56E-02 | AF | 0.0148 | | 0.032800 | 0.032300 | 3.10E-01 |
| rs10076365 | 5 | 161917408 | GABRA6 | AntiHTN | A | G | 0.8338 | -0.010038 | 0.003306 | 2.39E-03 | AF | 0.8126 | | -0.009800 | 0.008900 | 2.69E-01 |
| rs142415851 | 5 | 170016193 | GABRP | AntiHTN | T | C | 0.0302 | 0.016568 | 0.007513 | 2.74E-02 | AF | 0.0293 | | -0.007400 | 0.023400 | 7.52E-01 |
| rs79547497 | 5 | 171063750 | GABRP | AntiHTN | C | T | 0.0298 | 0.019308 | 0.007479 | 9.83E-03 | AF | 0.0260 | | 0.014100 | 0.025300 | 5.77E-01 |
| rs73318453 | 5 | 171095180 | GABRP | AntiHTN | G | T | 0.0215 | 0.022324 | 0.008440 | 8.17E-03 | AF | 0.0242 | | -0.010100 | 0.021500 | 6.41E-01 |
| rs76492099 | 7 | 81939205 | CACNA2D1 | AntiHTN | A | G | 0.0630 | -0.011911 | 0.005094 | 1.94E-02 | AF | 0.0570 | | -0.001100 | 0.014900 | 9.42E-01 |
| rs144549900 | 7 | 150244771 | KCNH2 | AntiHTN | C | A | 0.0662 | -0.012999 | 0.005023 | 9.66E-03 | AF | 0.0734 | | -0.022800 | 0.014200 | 1.10E-01 |
| rs2052130 | 7 | 150548598 | KCNH2 | AntiHTN | A | G | 0.1477 | 0.011114 | 0.003512 | 1.55E-03 | AF | 0.1527 | | -0.013900 | 0.009400 | 1.40E-01 |
| rs77185818 | 8 | 26288222 | ADRA1A | AntiHTN | A | G | 0.1200 | -0.008523 | 0.003794 | 2.47E-02 | AF | 0.1194 | | -0.001800 | 0.010700 | 8.63E-01 |
| rs184489 | 8 | 26305781 | ADRA1A | AntiHTN | C | T | 0.9822 | -0.019674 | 0.009273 | 3.39E-02 | AF | 0.9539 | | 0.009700 | 0.025500 | 7.04E-01 |
| rs1899494 | 8 | 26398643 | ADRA1A | AntiHTN | A | C | 0.2605 | 0.010537 | 0.002801 | 1.69E-04 | AF | 0.2745 | | 0.004600 | 0.007400 | 5.32E-01 |
| rs1383914 | 8 | 26723049 | ADRA1A | AntiHTN | C | T | 0.4944 | -0.006076 | 0.002453 | 1.33E-02 | AF | 0.4938 | | 0.010000 | 0.006600 | 1.31E-01 |
| rs35866749 | 8 | 38070128 | ADRB3 | AntiHTN | G | A | 0.1547 | -0.010636 | 0.003403 | 1.77E-03 | AF | 0.1485 | | -0.008300 | 0.010400 | 4.28E-01 |
| rs111617018 | 8 | 86420751 | CA1 | AntiHTN | A | G | 0.0687 | -0.011737 | 0.004873 | 1.60E-02 | AF | 0.0671 | | 0.001000 | 0.015700 | 9.47E-01 |
| rs62509890 | 8 | 87064009 | CA1 | AntiHTN | G | A | 0.1141 | 0.010297 | 0.003902 | 8.31E-03 | AF | 0.1137 | | -0.003700 | 0.010800 | 7.30E-01 |
| rs72686797 | 8 | 87215470 | CA2 | AntiHTN | C | A | 0.1017 | -0.010933 | 0.004254 | 1.02E-02 | AF | 0.1107 | | 0.012500 | 0.012000 | 3.00E-01 |
| rs2488161 | 10 | 18299365 | CACNB2 | AntiHTN | G | A | 0.1611 | 0.008202 | 0.003537 | 2.04E-02 | AF | 0.1582 | | 0.005800 | 0.009600 | 5.44E-01 |
| rs117926523 | 10 | 18336903 | CACNB2 | AntiHTN | G | A | 0.0159 | 0.029206 | 0.010161 | 4.05E-03 | AF | 0.0169 | | 0.043500 | 0.028900 | 1.32E-01 |
| rs2488158 | 10 | 18370324 | CACNB2 | AntiHTN | A | G | 0.1660 | 0.006929 | 0.003314 | 3.66E-02 | AF | 0.1606 | | 0.003300 | 0.008900 | 7.07E-01 |
| rs140766681 | 10 | 18409497 | CACNB2 | AntiHTN | C | A | 0.0212 | 0.021714 | 0.008551 | 1.11E-02 | AF | 0.0209 | | 0.031500 | 0.024400 | 1.98E-01 |
| rs10764322 | 10 | 18437061 | CACNB2 | AntiHTN | G | A | 0.3107 | 0.013826 | 0.002665 | 2.12E-07 | AF | 0.3050 | | 0.013800 | 0.007200 | 5.55E-02 |
| rs1757234 | 10 | 18526451 | CACNB2 | AntiHTN | A | G | 0.1164 | -0.007722 | 0.003844 | 4.46E-02 | AF | 0.1158 | | -0.035900 | 0.010400 | 5.81E-04 |
| rs2255266 | 10 | 18529720 | CACNB2 | AntiHTN | C | T | 0.7938 | -0.007116 | 0.003047 | 1.95E-02 | AF | 0.7733 | | -0.013400 | 0.008100 | 9.74E-02 |
| rs184067968 | 10 | 18529765 | CACNB2 | AntiHTN | A | C | 0.0165 | -0.024487 | 0.010073 | 1.51E-02 | AF | 0.0158 | | -0.002000 | 0.031400 | 9.49E-01 |
| rs12416030 | 10 | 18789075 | CACNB2 | AntiHTN | C | T | 0.2017 | 0.010977 | 0.003106 | 4.10E-04 | AF | 0.2092 | | 0.013600 | 0.008200 | 9.80E-02 |
| rs10828906 | 10 | 18841795 | CACNB2 | AntiHTN | T | C | 0.7351 | 0.011224 | 0.002791 | 5.79E-05 | AF | 0.7247 | | 0.009700 | 0.007600 | 1.98E-01 |
| rs115101482 | 10 | 18853351 | CACNB2 | AntiHTN | A | G | 0.0138 | 0.030768 | 0.010604 | 3.71E-03 | AF | 0.0159 | | 0.034600 | 0.030300 | 2.54E-01 |
| rs7901566 | 10 | 18987813 | CACNB2 | AntiHTN | A | G | 0.7378 | 0.007510 | 0.002821 | 7.76E-03 | AF | 0.7152 | | -0.005300 | 0.007600 | 4.86E-01 |
| rs12416245 | 10 | 111938114 | ADRA2A | AntiHTN | G | T | 0.1873 | 0.008922 | 0.003152 | 4.64E-03 | AF | 0.1827 | | 0.020600 | 0.008600 | 1.66E-02 |
| rs76100968 | 10 | 112887951 | ADRA2A | AntiHTN | T | C | 0.0628 | 0.010218 | 0.005060 | 4.35E-02 | AF | 0.0656 | | -0.004600 | 0.015300 | 7.62E-01 |
| rs6585052 | 10 | 113046403 | ADRA2A | AntiHTN | T | C | 0.9879 | 0.026214 | 0.011265 | 2.00E-02 | AF | 0.9516 | | 0.034000 | 0.026900 | 2.06E-01 |
| rs4366419 | 10 | 113049557 | ADRA2A | AntiHTN | G | A | 0.9188 | 0.012867 | 0.004513 | 4.36E-03 | AF | 0.8939 | | 0.019600 | 0.011900 | 1.01E-01 |
| rs4918689 | 10 | 113518004 | ADRA2A | AntiHTN | C | A | 0.0706 | 0.012865 | 0.004822 | 7.63E-03 | AF | 0.0746 | | -0.022000 | 0.012800 | 8.47E-02 |
| rs151591 | 10 | 115713566 | ADRB1 | AntiHTN | G | A | 0.7674 | 0.012450 | 0.002918 | 1.98E-05 | AF | 0.7555 | | 0.007600 | 0.007900 | 3.34E-01 |
| rs74717224 | 10 | 115719872 | ADRB1 | AntiHTN | T | G | 0.0586 | -0.014011 | 0.005245 | 7.55E-03 | AF | 0.0573 | | -0.005700 | 0.014400 | 6.93E-01 |
| rs11196589 | 10 | 115779717 | ADRB1 | AntiHTN | C | A | 0.4039 | 0.005045 | 0.002531 | 4.62E-02 | AF | 0.3995 | | 0.011600 | 0.006900 | 9.09E-02 |
| rs7076938 | 10 | 115789375 | ADRB1 | AntiHTN | T | C | 0.7350 | 0.017966 | 0.002794 | 1.28E-10 | AF | 0.7287 | | 0.010300 | 0.007500 | 1.71E-01 |
| rs7093444 | 10 | 115794324 | ADRB1 | AntiHTN | C | T | 0.1275 | -0.009607 | 0.003694 | 9.31E-03 | AF | 0.1368 | | -0.015000 | 0.009600 | 1.18E-01 |
| rs4072884 | 11 | 1482720 | TH | AntiHTN | T | C | 0.5900 | -0.005547 | 0.002513 | 2.73E-02 | AF | 0.5906 | | -0.002800 | 0.006900 | 6.84E-01 |
| rs71455916 | 11 | 2005794 | TH | AntiHTN | A | C | 0.1938 | 0.018705 | 0.003124 | 2.12E-09 | AF | 0.2037 | | 0.026800 | 0.009000 | 2.96E-03 |
| rs3842756 | 11 | 2180772 | TH | AntiHTN | T | C | 0.2274 | -0.006694 | 0.002935 | 2.26E-02 | AF | 0.2237 | | -0.011800 | 0.008200 | 1.52E-01 |
| rs11023983 | 11 | 16566923 | KCNJ11 | AntiHTN | C | A | 0.4700 | -0.007226 | 0.002462 | 3.33E-03 | AF | 0.4641 | | -0.008600 | 0.006700 | 1.97E-01 |
| rs11024048 | 11 | 16846310 | KCNJ11 | AntiHTN | C | T | 0.2665 | -0.008229 | 0.002789 | 3.18E-03 | AF | 0.2571 | | 0.000600 | 0.007800 | 9.36E-01 |
| rs2074311 | 11 | 17421860 | KCNJ11 | AntiHTN | G | A | 0.5858 | -0.009240 | 0.002495 | 2.13E-04 | AF | 0.5642 | | -0.002100 | 0.006700 | 7.57E-01 |
| rs10766408 | 11 | 17539212 | KCNJ11 | AntiHTN | C | T | 0.6240 | -0.005390 | 0.002543 | 3.40E-02 | AF | 0.6094 | | -0.000400 | 0.006800 | 9.57E-01 |
| rs12287965 | 11 | 17553706 | KCNJ11 | AntiHTN | T | C | 0.0813 | 0.010434 | 0.004489 | 2.01E-02 | AF | 0.0790 | | 0.007500 | 0.012400 | 5.48E-01 |
| rs7112615 | 11 | 68530318 | CPT1A | AntiHTN | A | C | 0.9384 | 0.011939 | 0.005123 | 1.98E-02 | AF | 0.9154 | | -0.018900 | 0.013900 | 1.74E-01 |
| rs117842198 | 11 | 68709890 | CPT1A | AntiHTN | A | G | 0.0097 | -0.026501 | 0.012663 | 3.64E-02 | AF | 0.0117 | | 0.073900 | 0.035800 | 3.93E-02 |
| rs7129324 | 11 | 68894909 | CPT1A | AntiHTN | A | G | 0.0166 | -0.023583 | 0.010238 | 2.12E-02 | AF | 0.0140 | | -0.048700 | 0.034200 | 1.55E-01 |
| rs11605129 | 11 | 69050915 | CPT1A | AntiHTN | T | C | 0.0528 | -0.019100 | 0.005508 | 5.25E-04 | AF | 0.0666 | | -0.022700 | 0.013700 | 9.72E-02 |
| rs73569213 | 11 | 128478380 | KCNJ1 | AntiHTN | A | G | 0.1779 | -0.006528 | 0.003213 | 4.22E-02 | AF | 0.1792 | | -0.000400 | 0.008800 | 9.64E-01 |
| rs882193 | 12 | 2350620 | CACNA1C | AntiHTN | A | G | 0.6183 | 0.006066 | 0.002579 | 1.87E-02 | AF | 0.6014 | | 0.006600 | 0.006900 | 3.41E-01 |
| rs55909860 | 12 | 2507848 | CACNA1C | AntiHTN | T | C | 0.0340 | 0.018480 | 0.006971 | 8.03E-03 | AF | 0.0352 | | 0.013600 | 0.020900 | 5.17E-01 |
| rs4765935 | 12 | 2563347 | CACNA1C | AntiHTN | C | A | 0.6986 | 0.005752 | 0.002709 | 3.37E-02 | AF | 0.6882 | | -0.013200 | 0.007500 | 7.70E-02 |
| rs2453467 | 12 | 49239951 | CACNB3 | AntiHTN | T | C | 0.7883 | 0.006459 | 0.003034 | 3.33E-02 | AF | 0.7721 | | -0.002200 | 0.008500 | 7.94E-01 |
| rs12317778 | 12 | 50105637 | CACNB3 | AntiHTN | C | T | 0.0823 | -0.021217 | 0.004493 | 2.34E-06 | AF | 0.0794 | | -0.029000 | 0.012300 | 1.78E-02 |
| rs80339013 | 15 | 26272751 | GABRA5 | AntiHTN | C | T | 0.1174 | 0.012881 | 0.003896 | 9.47E-04 | AF | 0.1181 | | -0.003000 | 0.010900 | 7.84E-01 |
| rs8030011 | 15 | 26818362 | GABRB3 | AntiHTN | A | G | 0.1292 | -0.008237 | 0.003677 | 2.51E-02 | AF | 0.1268 | | 0.011800 | 0.009900 | 2.34E-01 |
| rs117280013 | 15 | 26969128 | GABRG3 | AntiHTN | A | G | 0.0431 | -0.012372 | 0.006068 | 4.15E-02 | AF | 0.0539 | | 0.001800 | 0.016800 | 9.16E-01 |
| rs140443467 | 15 | 27722954 | GABRG3 | AntiHTN | G | A | 0.0281 | -0.029713 | 0.007696 | 1.13E-04 | AF | 0.0318 | | 0.007800 | 0.020500 | 7.02E-01 |
| rs12914000 | 15 | 47906718 | SLC12A1 | AntiHTN | C | T | 0.1631 | -0.006925 | 0.003383 | 4.07E-02 | AF | 0.1569 | | 0.000100 | 0.009400 | 9.89E-01 |
| rs71380229 | 16 | 1034503 | CACNA1H | AntiHTN | C | A | 0.0791 | 0.010421 | 0.004584 | 2.30E-02 | AF | 0.0914 | | 0.004300 | 0.012500 | 7.31E-01 |
| rs12926678 | 16 | 1187931 | CACNA1H | AntiHTN | T | C | 0.3830 | -0.005138 | 0.002575 | 4.60E-02 | AF | 0.3715 | | -0.002300 | 0.007500 | 7.59E-01 |
| rs117177120 | 16 | 1238875 | CACNA1H | AntiHTN | G | T | 0.0573 | 0.018092 | 0.005430 | 8.63E-04 | AF | 0.0676 | | 0.007200 | 0.015700 | 6.48E-01 |
| rs4347630 | 16 | 1243557 | CACNA1H | AntiHTN | C | T | 0.8154 | 0.007868 | 0.003295 | 1.70E-02 | AF | 0.8104 | | -0.016100 | 0.010300 | 1.16E-01 |
| rs8053994 | 16 | 1244201 | CACNA1H | AntiHTN | A | G | 0.5550 | -0.006127 | 0.002537 | 1.57E-02 | AF | 0.5449 | | -0.013400 | 0.007400 | 6.86E-02 |
| rs1977100 | 16 | 1310921 | CACNA1H | AntiHTN | G | A | 0.3203 | -0.006019 | 0.002638 | 2.25E-02 | AF | 0.3283 | | -0.011800 | 0.007400 | 1.14E-01 |
| rs150274214 | 16 | 22727592 | SCNN1G | AntiHTN | A | G | 0.0193 | 0.023979 | 0.009202 | 9.16E-03 | AF | 0.0174 | | -0.018800 | 0.032100 | 5.59E-01 |
| rs9931863 | 16 | 23249779 | SCNN1B | AntiHTN | G | A | 0.2436 | 0.006084 | 0.002892 | 3.54E-02 | AF | 0.2572 | | -0.008900 | 0.007900 | 2.61E-01 |
| rs2520014 | 16 | 23794098 | SCNN1B | AntiHTN | A | G | 0.4266 | -0.005332 | 0.002487 | 3.20E-02 | AF | 0.4266 | | 0.003500 | 0.006700 | 6.03E-01 |
| rs2338115 | 17 | 36929578 | CACNB1 | AntiHTN | T | C | 0.5544 | -0.005892 | 0.002476 | 1.73E-02 | AF | 0.5363 | | 0.004500 | 0.006200 | 4.64E-01 |
| rs71369724 | 17 | 37326350 | CACNB1 | AntiHTN | A | G | 0.0540 | 0.011835 | 0.005465 | 3.03E-02 | AF | 0.0507 | | -0.016000 | 0.017400 | 3.59E-01 |
| rs61554907 | 17 | 38220432 | CACNB1 | AntiHTN | T | G | 0.1095 | 0.008199 | 0.003974 | 3.91E-02 | AF | 0.1185 | | 0.025900 | 0.010600 | 1.47E-02 |
| rs731152 | 17 | 40279680 | AOC3 | AntiHTN | A | G | 0.2268 | -0.006931 | 0.002934 | 1.82E-02 | AF | 0.2322 | | 0.015000 | 0.007800 | 5.50E-02 |
| rs138643143 | 17 | 40709867 | AOC3 | AntiHTN | A | G | 0.0750 | 0.019321 | 0.004962 | 9.89E-05 | AF | 0.0747 | | 0.027800 | 0.015100 | 6.68E-02 |
| rs74754758 | 17 | 40902035 | AOC3 | AntiHTN | C | T | 0.0170 | -0.035157 | 0.009820 | 3.44E-04 | AF | 0.0183 | | -0.025600 | 0.028000 | 3.62E-01 |
| rs324075 | 17 | 41026523 | AOC3 | AntiHTN | G | A | 0.1878 | -0.007210 | 0.003185 | 2.36E-02 | AF | 0.1845 | | 0.000800 | 0.008900 | 9.25E-01 |
| rs74835612 | 17 | 41760775 | AOC3 | AntiHTN | A | G | 0.0623 | 0.017691 | 0.005062 | 4.74E-04 | AF | 0.0621 | | -0.019900 | 0.014600 | 1.73E-01 |
| rs8065903 | 17 | 48629458 | CACNA1G | AntiHTN | G | A | 0.7381 | -0.007571 | 0.002795 | 6.76E-03 | AF | 0.7218 | | -0.015300 | 0.007600 | 4.29E-02 |
| rs198535 | 17 | 48635052 | CACNA1G | AntiHTN | G | A | 0.4177 | -0.011022 | 0.002506 | 1.10E-05 | AF | 0.4239 | | -0.005900 | 0.006800 | 3.85E-01 |
| rs118166304 | 17 | 48951566 | CACNA1G | AntiHTN | G | T | 0.0177 | -0.019382 | 0.009370 | 3.86E-02 | AF | 0.0157 | | 0.095100 | 0.031100 | 2.25E-03 |
| rs34157595 | 17 | 49200947 | CACNA1G | AntiHTN | C | T | 0.3950 | -0.005087 | 0.002532 | 4.45E-02 | AF | 0.4041 | | -0.001400 | 0.007000 | 8.40E-01 |
| rs4968783 | 17 | 61550729 | ACE | AntiHTN | A | C | 0.6194 | -0.009012 | 0.002544 | 3.97E-04 | AF | 0.5955 | | 0.001900 | 0.006800 | 7.84E-01 |
| rs78784512 | 17 | 64195835 | CACNG1 | AntiHTN | T | C | 0.0228 | 0.016976 | 0.008618 | 4.89E-02 | AF | 0.0215 | | -0.012200 | 0.026900 | 6.50E-01 |
| rs2241359 | 19 | 14586245 | PTGER1 | AntiHTN | A | G | 0.1615 | 0.007838 | 0.003344 | 1.91E-02 | AF | 0.1608 | | 0.005200 | 0.009300 | 5.80E-01 |
| rs3760702 | 19 | 14588237 | PTGER1 | AntiHTN | A | G | 0.3302 | -0.005445 | 0.002617 | 3.75E-02 | AF | 0.3221 | | -0.007900 | 0.007400 | 2.90E-01 |
| rs62111755 | 19 | 46253766 | PTGIR | AntiHTN | G | T | 0.1523 | 0.008838 | 0.003415 | 9.66E-03 | AF | 0.1578 | | 0.005500 | 0.009100 | 5.44E-01 |
| rs138809535 | 19 | 46279757 | PTGIR | AntiHTN | T | C | 0.0192 | 0.017792 | 0.008998 | 4.80E-02 | AF | 0.0186 | | 0.002300 | 0.027400 | 9.34E-01 |
| rs117687892 | 19 | 46572541 | PTGIR | AntiHTN | T | C | 0.0358 | -0.014775 | 0.006737 | 2.83E-02 | AF | 0.0355 | | 0.005700 | 0.019700 | 7.71E-01 |
| rs735710 | 20 | 3692839 | ADRA1D | AntiHTN | C | T | 0.0704 | -0.014293 | 0.004855 | 3.24E-03 | AF | 0.0727 | | -0.003200 | 0.013000 | 8.07E-01 |
| rs4815605 | 20 | 3777946 | ADRA1D | AntiHTN | A | G | 0.5567 | -0.005284 | 0.002482 | 3.32E-02 | AF | 0.5734 | | 0.000300 | 0.006900 | 9.66E-01 |
| rs55778060 | 20 | 4189771 | ADRA1D | AntiHTN | T | C | 0.1250 | 0.010754 | 0.003810 | 4.76E-03 | AF | 0.1223 | | 0.006500 | 0.010700 | 5.45E-01 |
| rs57698607 | 20 | 4438261 | ADRA1D | AntiHTN | T | C | 0.0575 | 0.014320 | 0.005309 | 7.00E-03 | AF | 0.0552 | | -0.002200 | 0.014900 | 8.82E-01 |
| rs2903908 | 20 | 44693947 | SLC12A5 | AntiHTN | C | T | 0.2605 | 0.007690 | 0.002811 | 6.23E-03 | AF | 0.2648 | | -0.012000 | 0.007600 | 1.14E-01 |
| rs136832 | 22 | 40046538 | CACNA1I | AntiHTN | T | C | 0.1864 | 0.006252 | 0.003158 | 4.77E-02 | AF | 0.1896 | | 0.011100 | 0.008600 | 1.94E-01 |
| rs117741951 | 22 | 40403113 | CACNA1I | AntiHTN | T | C | 0.0294 | -0.018231 | 0.007466 | 1.46E-02 | AF | 0.0291 | | 0.017500 | 0.023800 | 4.62E-01 |

**Abbreviations:** AF, atrial fibrillation; AntiHTN; all 12 antihypertensive drug classes combined; Chr, chromosome; EA, effect allele; EAF, effect allele frequency; OA, other allele; Pos, genomic position; Pval, p-value; SE, standard error; SNP, single nucleotide polymorphism.

**Table S19. Effect estimates for the associations of the genetic variants with angiotensin-converting enzyme inhibitors and atrial fibrillation for the secondary analyses**

|  | | | | **Exposure effect estimates** | | | | | | | | | **Outcome effect estimates** | | | |
| --- | --- | --- | --- | --- | --- | --- | --- | --- | --- | --- | --- | --- | --- | --- | --- | --- |
| **SNP** | **Chr** | **Pos** | **Gene** | **Trait** | **EA** | **OA** | **EAF** | **Beta** | **SE** | **Pval** | **Trait** | **EAF** | | **Beta** | **SE** | **Pval** |
| rs4291 | 17 | 61554194 | ACE | ACEIs | A | T | 0.6155 | -0.283900 | 0.031200 | 8.65E-20 | AF | 0.6016 | | 0.003100 | 0.006800 | 6.54E-01 |

**Abbreviations:** ACEIs, angiotensin-converting enzyme inhibitors; AF, atrial fibrillation; Chr, chromosome; EA, effect allele; EAF, effect allele frequency; OA, other allele; Pos, genomic position; Pval, p-value; SE, standard error; SNP, single nucleotide polymorphism.

**Table S20. Effect estimates for the associations of the genetic variants with beta-adrenoceptor blockers and atrial fibrillation for the secondary analyses**

|  | | | | **Exposure effect estimates** | | | | | | | | | **Outcome effect estimates** | | | |
| --- | --- | --- | --- | --- | --- | --- | --- | --- | --- | --- | --- | --- | --- | --- | --- | --- |
| **SNP** | **Chr** | **Pos** | **Gene** | **Trait** | **EA** | **OA** | **EAF** | **Beta** | **SE** | **Pval** | **Trait** | **EAF** | | **Beta** | **SE** | **Pval** |
| rs11196549 | 10 | 115707298 |  | BBs | A | G | 0.0425 | 0.688400 | 0.078400 | 1.58E-18 | AF | 0.0441 | | 0.042700 | 0.018400 | 2.06E-02 |
| rs460718 | 10 | 115721364 |  | BBs | A | G | 0.3266 | -0.276400 | 0.032400 | 1.36E-17 | AF | 0.3333 | | -0.010000 | 0.007100 | 1.62E-01 |
| rs11196597 | 10 | 115788094 |  | BBs | A | G | 0.1330 | 0.285800 | 0.045800 | 4.23E-10 | AF | 0.1431 | | 0.012300 | 0.009700 | 2.05E-01 |
| rs17875473 | 10 | 115800294 |  | BBs | T | C | 0.0871 | 0.328300 | 0.055200 | 2.66E-09 | AF | 0.0839 | | 0.013500 | 0.012300 | 2.71E-01 |
| rs1801253 | 10 | 115805056 |  | BBs | C | G | 0.7338 | 0.462600 | 0.034400 | 2.84E-41 | AF | 0.7308 | | 0.011600 | 0.007600 | 1.29E-01 |
| rs4359161 | 10 | 115826508 |  | BBs | A | G | 0.1812 | -0.266200 | 0.039100 | 9.46E-12 | AF | 0.1865 | | -0.010900 | 0.008500 | 2.00E-01 |

**Abbreviations:** AF, atrial fibrillation; BBs, beta-adrenoceptor blockers; Chr, chromosome; EA, effect allele; EAF, effect allele frequency; OA, other allele; Pos, genomic position; Pval, p-value; SE, standard error; SNP, single nucleotide polymorphism.

**Table S21. Effect estimates for the associations of the genetic variants with calcium channel blockers and atrial fibrillation for the secondary analyses**

|  | | | | **Exposure effect estimates** | | | | | | | | | **Outcome effect estimates** | | | |
| --- | --- | --- | --- | --- | --- | --- | --- | --- | --- | --- | --- | --- | --- | --- | --- | --- |
| **SNP** | **Chr** | **Pos** | **Gene** | **Trait** | **EA** | **OA** | **EAF** | **Beta** | **SE** | **Pval** | **Trait** | **EAF** | | **Beta** | **SE** | **Pval** |
| rs3821843 | 3 | 53558012 |  | CCBs | A | G | 0.6808 | 0.337300 | 0.033500 | 6.56E-24 | AF | 0.6737 | | 0.023000 | 0.007400 | 1.89E-03 |
| rs114987861 | 3 | 53605712 |  | CCBs | A | G | 0.0284 | 0.528900 | 0.095800 | 3.36E-08 | AF | 0.0312 | | 0.011300 | 0.020900 | 5.88E-01 |
| rs113210396 | 3 | 53612327 |  | CCBs | T | G | 0.0451 | -0.433800 | 0.077000 | 1.76E-08 | AF | 0.0478 | | -0.028300 | 0.017300 | 1.01E-01 |
| rs7340705 | 3 | 53734443 |  | CCBs | T | C | 0.6732 | -0.242500 | 0.032200 | 4.87E-14 | AF | 0.6643 | | -0.017400 | 0.007000 | 1.29E-02 |
| rs2488136 | 10 | 18334521 |  | CCBs | A | G | 0.2875 | 0.226100 | 0.033400 | 1.22E-11 | AF | 0.3034 | | 0.020900 | 0.007300 | 4.23E-03 |
| rs1888693 | 10 | 18440444 |  | CCBs | A | G | 0.3449 | 0.385800 | 0.031700 | 4.69E-34 | AF | 0.3349 | | 0.004900 | 0.007000 | 4.86E-01 |
| rs16916914 | 10 | 18457722 |  | CCBs | T | C | 0.9631 | -0.563600 | 0.080600 | 2.72E-12 | AF | 0.9637 | | -0.013600 | 0.018200 | 4.56E-01 |
| rs7076319 | 10 | 18459450 |  | CCBs | A | G | 0.7339 | -0.321000 | 0.034100 | 5.07E-21 | AF | 0.7387 | | -0.015900 | 0.007500 | 3.40E-02 |
| rs61278674 | 10 | 18481737 |  | CCBs | A | G | 0.9062 | -0.329800 | 0.054000 | 1.03E-09 | AF | 0.8984 | | -0.014300 | 0.011600 | 2.20E-01 |
| rs1779209 | 10 | 18514561 |  | CCBs | T | C | 0.2876 | 0.273600 | 0.033600 | 4.23E-16 | AF | 0.2866 | | 0.013300 | 0.007400 | 7.18E-02 |
| rs10828399 | 10 | 18553968 |  | CCBs | A | G | 0.5218 | -0.194700 | 0.030200 | 1.10E-10 | AF | 0.5155 | | 0.004100 | 0.006600 | 5.40E-01 |
| rs10828452 | 10 | 18592450 |  | CCBs | A | T | 0.7930 | 0.304600 | 0.038800 | 4.20E-15 | AF | 0.7976 | | 0.016400 | 0.008600 | 5.73E-02 |
| rs10828542 | 10 | 18627285 |  | CCBs | A | G | 0.6137 | 0.181700 | 0.031100 | 5.18E-09 | AF | 0.6293 | | 0.013600 | 0.006900 | 4.80E-02 |
| rs12780039 | 10 | 18678987 |  | CCBs | C | G | 0.1210 | 0.285200 | 0.047000 | 1.26E-09 | AF | 0.1326 | | 0.000900 | 0.010100 | 9.30E-01 |
| rs112133583 | 10 | 18695681 |  | CCBs | T | C | 0.0299 | -0.554600 | 0.097300 | 1.18E-08 | AF | 0.0315 | | -0.049300 | 0.022000 | 2.51E-02 |
| rs11014170 | 10 | 18710991 |  | CCBs | A | G | 0.0206 | -0.670100 | 0.115000 | 5.61E-09 | AF | 0.0185 | | -0.035700 | 0.027900 | 2.01E-01 |
| rs7923191 | 10 | 18727901 |  | CCBs | A | G | 0.7918 | -0.369000 | 0.037600 | 1.10E-22 | AF | 0.7836 | | -0.023600 | 0.008100 | 3.61E-03 |
| rs12258967 | 10 | 18727959 |  | CCBs | C | G | 0.7047 | 0.632700 | 0.033700 | 1.08E-78 | AF | 0.7162 | | 0.030500 | 0.007600 | 5.38E-05 |
| rs72786098 | 10 | 18729855 |  | CCBs | A | G | 0.0322 | -0.503300 | 0.088300 | 1.18E-08 | AF | 0.0335 | | 0.000600 | 0.020200 | 9.76E-01 |
| rs1998822 | 10 | 18755664 |  | CCBs | A | G | 0.7234 | -0.195800 | 0.034300 | 1.15E-08 | AF | 0.7189 | | -0.012700 | 0.007400 | 8.62E-02 |
| rs4748474 | 10 | 18790727 |  | CCBs | A | G | 0.5214 | 0.194600 | 0.030400 | 1.61E-10 | AF | 0.5200 | | 0.015000 | 0.006600 | 2.38E-02 |
| rs2239046 | 12 | 2434419 |  | CCBs | A | G | 0.6817 | 0.208200 | 0.032200 | 9.58E-11 | AF | 0.6605 | | 0.011800 | 0.007100 | 9.61E-02 |
| rs714277 | 12 | 2514270 |  | CCBs | T | C | 0.2834 | 0.198600 | 0.033300 | 2.38E-09 | AF | 0.2719 | | 0.010900 | 0.007500 | 1.48E-01 |
| rs150857355 | 12 | 49209340 |  | CCBs | C | G | 0.0217 | 0.940600 | 0.112200 | 5.20E-17 | AF | 0.0219 | | 0.043700 | 0.026200 | 9.58E-02 |

**Abbreviations:** AF, atrial fibrillation; CCBs, calcium channel blockers; Chr, chromosome; EA, effect allele; EAF, effect allele frequency; OA, other allele; Pos, genomic position; Pval, p-value; SE, standard error; SNP, single nucleotide polymorphism.

**Table S22. Effect estimates for the associations of the genetic variants with all 3 antihypertensive drug classes combined and atrial fibrillation for the secondary analyses**

|  | | | | **Exposure effect estimates** | | | | | | | | | **Outcome effect estimates** | | | |
| --- | --- | --- | --- | --- | --- | --- | --- | --- | --- | --- | --- | --- | --- | --- | --- | --- |
| **SNP** | **Chr** | **Pos** | **Gene** | **Trait** | **EA** | **OA** | **EAF** | **Beta** | **SE** | **Pval** | **Trait** | **EAF** | | **Beta** | **SE** | **Pval** |
| rs3821843 | 3 | 53558012 |  | AntiHTN | A | G | 0.6808 | 0.337300 | 0.033500 | 6.56E-24 | AF | 0.6737 | | 0.023000 | 0.007400 | 1.89E-03 |
| rs114987861 | 3 | 53605712 |  | AntiHTN | A | G | 0.0284 | 0.528900 | 0.095800 | 3.36E-08 | AF | 0.0312 | | 0.011300 | 0.020900 | 5.88E-01 |
| rs113210396 | 3 | 53612327 |  | AntiHTN | T | G | 0.0451 | -0.433800 | 0.077000 | 1.76E-08 | AF | 0.0478 | | -0.028300 | 0.017300 | 1.01E-01 |
| rs7340705 | 3 | 53734443 |  | AntiHTN | T | C | 0.6732 | -0.242500 | 0.032200 | 4.87E-14 | AF | 0.6643 | | -0.017400 | 0.007000 | 1.29E-02 |
| rs2488136 | 10 | 18334521 |  | AntiHTN | A | G | 0.2875 | 0.226100 | 0.033400 | 1.22E-11 | AF | 0.3034 | | 0.020900 | 0.007300 | 4.23E-03 |
| rs1888693 | 10 | 18440444 |  | AntiHTN | A | G | 0.3449 | 0.385800 | 0.031700 | 4.69E-34 | AF | 0.3349 | | 0.004900 | 0.007000 | 4.86E-01 |
| rs16916914 | 10 | 18457722 |  | AntiHTN | T | C | 0.9631 | -0.563600 | 0.080600 | 2.72E-12 | AF | 0.9637 | | -0.013600 | 0.018200 | 4.56E-01 |
| rs7076319 | 10 | 18459450 |  | AntiHTN | A | G | 0.7339 | -0.321000 | 0.034100 | 5.07E-21 | AF | 0.7387 | | -0.015900 | 0.007500 | 3.40E-02 |
| rs61278674 | 10 | 18481737 |  | AntiHTN | A | G | 0.9062 | -0.329800 | 0.054000 | 1.03E-09 | AF | 0.8984 | | -0.014300 | 0.011600 | 2.20E-01 |
| rs1779209 | 10 | 18514561 |  | AntiHTN | T | C | 0.2876 | 0.273600 | 0.033600 | 4.23E-16 | AF | 0.2866 | | 0.013300 | 0.007400 | 7.18E-02 |
| rs10828399 | 10 | 18553968 |  | AntiHTN | A | G | 0.5218 | -0.194700 | 0.030200 | 1.10E-10 | AF | 0.5155 | | 0.004100 | 0.006600 | 5.40E-01 |
| rs10828452 | 10 | 18592450 |  | AntiHTN | A | T | 0.7930 | 0.304600 | 0.038800 | 4.20E-15 | AF | 0.7976 | | 0.016400 | 0.008600 | 5.73E-02 |
| rs10828542 | 10 | 18627285 |  | AntiHTN | A | G | 0.6137 | 0.181700 | 0.031100 | 5.18E-09 | AF | 0.6293 | | 0.013600 | 0.006900 | 4.80E-02 |
| rs12780039 | 10 | 18678987 |  | AntiHTN | C | G | 0.1210 | 0.285200 | 0.047000 | 1.26E-09 | AF | 0.1326 | | 0.000900 | 0.010100 | 9.30E-01 |
| rs112133583 | 10 | 18695681 |  | AntiHTN | T | C | 0.0299 | -0.554600 | 0.097300 | 1.18E-08 | AF | 0.0315 | | -0.049300 | 0.022000 | 2.51E-02 |
| rs11014170 | 10 | 18710991 |  | AntiHTN | A | G | 0.0206 | -0.670100 | 0.115000 | 5.61E-09 | AF | 0.0185 | | -0.035700 | 0.027900 | 2.01E-01 |
| rs7923191 | 10 | 18727901 |  | AntiHTN | A | G | 0.7918 | -0.369000 | 0.037600 | 1.10E-22 | AF | 0.7836 | | -0.023600 | 0.008100 | 3.61E-03 |
| rs12258967 | 10 | 18727959 |  | AntiHTN | C | G | 0.7047 | 0.632700 | 0.033700 | 1.08E-78 | AF | 0.7162 | | 0.030500 | 0.007600 | 5.38E-05 |
| rs72786098 | 10 | 18729855 |  | AntiHTN | A | G | 0.0322 | -0.503300 | 0.088300 | 1.18E-08 | AF | 0.0335 | | 0.000600 | 0.020200 | 9.76E-01 |
| rs1998822 | 10 | 18755664 |  | AntiHTN | A | G | 0.7234 | -0.195800 | 0.034300 | 1.15E-08 | AF | 0.7189 | | -0.012700 | 0.007400 | 8.62E-02 |
| rs4748474 | 10 | 18790727 |  | AntiHTN | A | G | 0.5214 | 0.194600 | 0.030400 | 1.61E-10 | AF | 0.5200 | | 0.015000 | 0.006600 | 2.38E-02 |
| rs11196549 | 10 | 115707298 |  | AntiHTN | A | G | 0.0425 | 0.688400 | 0.078400 | 1.58E-18 | AF | 0.0441 | | 0.042700 | 0.018400 | 2.06E-02 |
| rs460718 | 10 | 115721364 |  | AntiHTN | A | G | 0.3266 | -0.276400 | 0.032400 | 1.36E-17 | AF | 0.3333 | | -0.010000 | 0.007100 | 1.62E-01 |
| rs11196597 | 10 | 115788094 |  | AntiHTN | A | G | 0.1330 | 0.285800 | 0.045800 | 4.23E-10 | AF | 0.1431 | | 0.012300 | 0.009700 | 2.05E-01 |
| rs17875473 | 10 | 115800294 |  | AntiHTN | T | C | 0.0871 | 0.328300 | 0.055200 | 2.66E-09 | AF | 0.0839 | | 0.013500 | 0.012300 | 2.71E-01 |
| rs1801253 | 10 | 115805056 |  | AntiHTN | C | G | 0.7338 | 0.462600 | 0.034400 | 2.84E-41 | AF | 0.7308 | | 0.011600 | 0.007600 | 1.29E-01 |
| rs4359161 | 10 | 115826508 |  | AntiHTN | A | G | 0.1812 | -0.266200 | 0.039100 | 9.46E-12 | AF | 0.1865 | | -0.010900 | 0.008500 | 2.00E-01 |
| rs2239046 | 12 | 2434419 |  | AntiHTN | A | G | 0.6817 | 0.208200 | 0.032200 | 9.58E-11 | AF | 0.6605 | | 0.011800 | 0.007100 | 9.61E-02 |
| rs714277 | 12 | 2514270 |  | AntiHTN | T | C | 0.2834 | 0.198600 | 0.033300 | 2.38E-09 | AF | 0.2719 | | 0.010900 | 0.007500 | 1.48E-01 |
| rs150857355 | 12 | 49209340 |  | AntiHTN | C | G | 0.0217 | 0.940600 | 0.112200 | 5.20E-17 | AF | 0.0219 | | 0.043700 | 0.026200 | 9.58E-02 |
| rs4291 | 17 | 61554194 |  | AntiHTN | A | T | 0.6155 | -0.283900 | 0.031200 | 8.65E-20 | AF | 0.6016 | | 0.003100 | 0.006800 | 6.54E-01 |

**Abbreviations:** AF, atrial fibrillation; AntiHTN; all 12 antihypertensive drug classes combined; Chr, chromosome; EA, effect allele; EAF, effect allele frequency; OA, other allele; Pos, genomic position; Pval, p-value; SE, standard error; SNP, single nucleotide polymorphism.

**Figure S1. Flow chart for selection of genetic variants for the secondary analyses**

**
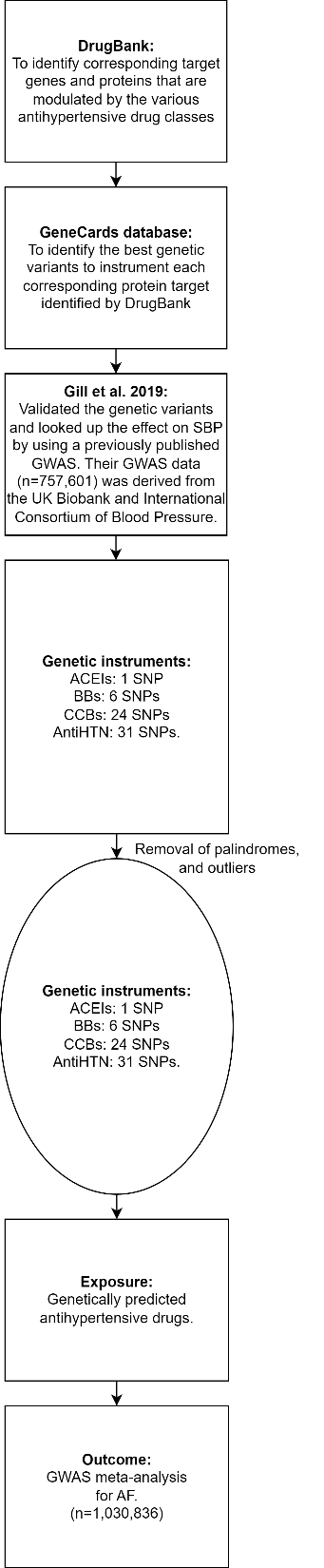
**

**Abbreviations:** AABs, adrenergic neuron blockers; ACEIs, angiotensin-converting enzyme inhibitors; AF, atrial fibrillation; ANBs, alpha adrenocepter blockers; AntiHTN; all 3 antihypertensive drug classes combined; ARBs, angiotensin-II receptor antagonists; BBs, beta-adrenoceptor blockers; CAAHTN, centrally acting antihypertensives; CCBs, calcium channel blockers; GWAS, genome wide association study; LDs, loop diuretics; MRAs, mineralocorticoid receptor antagonists; n, number; PSDs, potassium sparing diuretics and aldosterone antagonists; RIs, renin inhibitors; SBP, systolic blood pressure; SNP(s), single nucleotide polymorphism(s); Thiazides; thiazides and related diuretics; VDs, vasodilators.


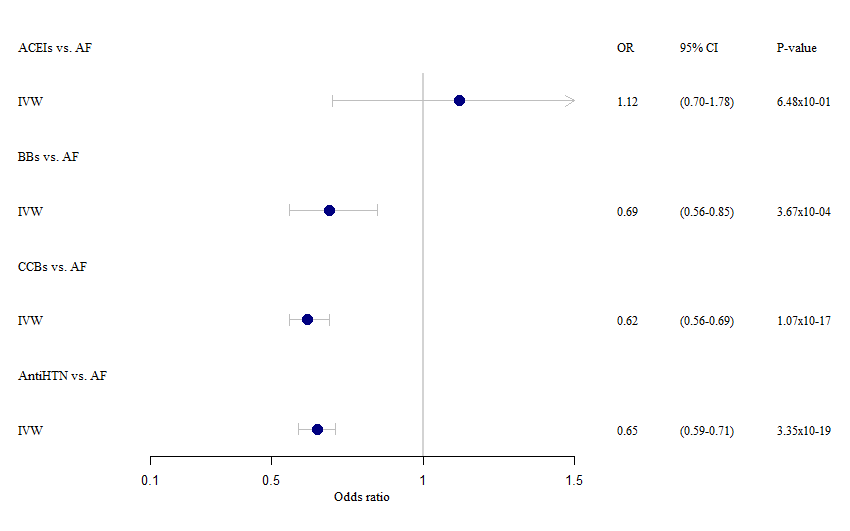
**Figure S2. Forest plot which visualizes the Mendelian randomization analyses between antihypertensive drug classes and atrial fibrillation for the secondary analyses


Abbreviations:** AABs, adrenergic neuron blockers; ACEIs, angiotensin-converting enzyme inhibitors; AF, atrial fibrillation; ANBs, alpha adrenocepter blockers; AntiHTN; all 3 antihypertensive drug classes combined; ARBs, angiotensin-II receptor antagonists; BBs, beta-adrenoceptor blockers; CAAHTN, centrally acting antihypertensives; CCBs, calcium channel blockers; CI, confidence interval; IVW, inverse variance weighted; LDs, loop diuretics; MRAs; mineralocorticoid receptor antagonists; odds ratio; PSDs, potassium sparing diuretics and aldosterone antagonists; RIs; renin inhibitors; Thiazides; thiazides and related diuretics; VDs, vasodilators.

Odds ratios represent a genetically determined 10 unit decrease of ln(SBP) through the various antihypertensive drug classes with the odds of atrial fibrillation.
