# Supplementary material for: Antihypertensive Drugs for the Prevention of Atrial Fibrillation: A Drug Target Mendelian Randomization Study
Source: Hypertension. 2024 Jun 19;81(8):1766–75. doi: 10.1161/HYPERTENSIONAHA.123.21858 (PMC11251507; doi:10.1161/HYPERTENSIONAHA.123.21858)
Supplement: Supplementary file 2 [file hyp-81-1766-s002.pdf]

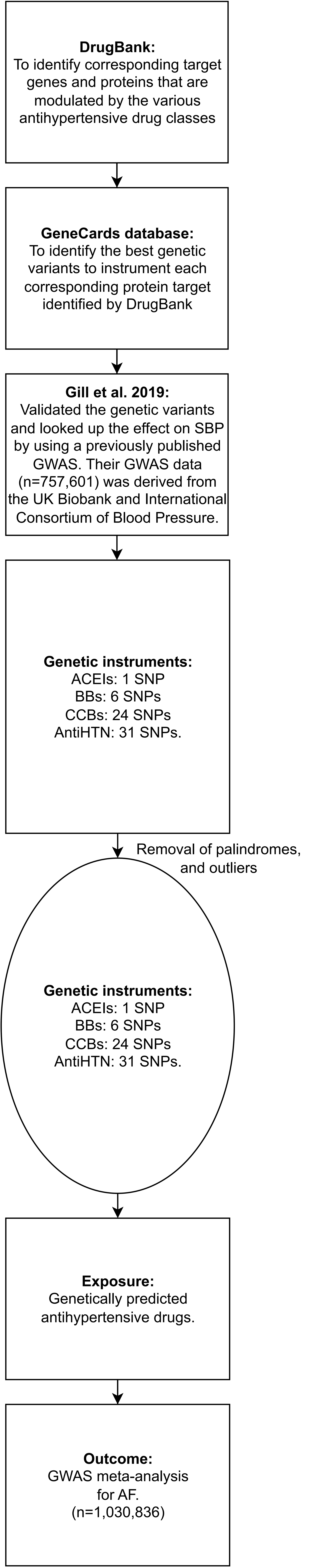
DrugBank:
To identify corresponding target genes and proteins that are modulated by the various antihypertensive drug classes
GeneCards database:
To identify the best genetic variants to instrument each corresponding protein target identified by DrugBank
Gill et al. 2019:
Validated the genetic variants and looked up the effect on SBP by using a previously published GWAS. Their GWAS data (n=757,601) was derived from the UK Biobank and International Consortium of Blood Pressure.
Genetic instruments:
ACEIs: 1 SNP
BBs: 6 SNPs
CCBs: 24 SNPs
AntiHTN: 31 SNPs.
Removal of palindromes, and outliers
Genetic instruments:
ACEIs: 1 SNP
BBs: 6 SNPs
CCBs: 24 SNPs
AntiHTN: 31 SNPs.
Exposure:
Genetically predicted antihypertensive drugs.
Outcome:
GWAS meta-analysis for AF.
(n=1,030,836)
